# Edoxaban for Stroke Prevention in Atrial Fibrillation in Spain and Portugal: 4-Year Follow-Up of the Observational ETNA-AF-Europe Study

**DOI:** 10.3390/jcm15114085

**Published:** 2026-05-25

**Authors:** Gonzalo Barón-Esquivias, Pedro Monteiro, José Luis Santiago-Ruiz, Lucía Basterra-Trincado, Luís Vieira dos Santos, Jorge Ruivo, Eva-Maria Fronk, Paulus Kirchhof, Raffaele De Caterina

**Affiliations:** 1 Cardiology Department, Virgen del Rocío University Hospital, 41013 Seville, Spain; 2 Cardiology Department, Coimbra University Hospital and Medical School, 3046-853 Coimbra, Portugal; pedromontei@gmail.com; 3 Medical Department, Daiichi Sankyo Spain, 28045 Madrid, Spain; jose.santiago@daiichisankyo.com (J.L.S.-R.); lucia.basterra@daiichisankyo.com (L.B.-T.); 4 Medical Department, Daiichi Sankyo Portugal, 2740-257 Porto Salvo, Portugal; luis.santos@daiichisankyo.com (L.V.d.S.); jorge.ruivo@daiichisankyo.com (J.R.); 5 Data and Statistical Sciences—Evidence Generation Department, Daiichi Sankyo, 81379 Munich, Germany; eva-maria.fronk@daiichisankyo.com; 6 Cardiology Department, University Heart and Vascular Center Hamburg, 20246 Hamburg, Germany; p.kirchhof@uke.de; 7 German Center for Cardiovascular Research (DZHK), Partner Site North, 20246 Hamburg, Germany; 8 Institute of Cardiovascular Sciences, University of Birmingham, Birmingham B15 2TT, UK; 9 Department of Surgical, Medical, Molecular Pathology and Critical Sciences, University of Pisa, 56126 Pisa, Italy; raffaele.decaterina@unipi.it; 10 Cardiovascular Division, Pisa University Hospital, 56126 Pisa, Italy

**Keywords:** edoxaban, atrial fibrillation, observational study, post-authorization study, non-vitamin K antagonist oral anticoagulant, stroke prevention, routine care, Spain, Portugal

## Abstract

**Background/Objectives:** Edoxaban is a non-vitamin K antagonist oral anticoagulant indicated in patients with non-valvular atrial fibrillation to prevent stroke and systemic embolism. The objective was to obtain real-world data on the safety and effectiveness of edoxaban in routine care among patients with atrial fibrillation. **Methods:** ETNA-AF-Europe was a multinational, multicenter, prospective, post-authorization observational study. Its objectives were to assess the real-world safety of edoxaban (bleeding events and mortality) and its effectiveness by evaluating stroke, systemic embolic events, and cardiovascular events over 4 years. This article presents the results for Spain and Portugal. **Results:** In Spain and Portugal, 931 patients were included in the full analysis set. At baseline, 75.3% of patients were receiving edoxaban 60 mg, and 23.3% were receiving edoxaban 30 mg. Participants receiving 30 mg were older, more likely to be frail, and had a higher prevalence of comorbidities. The annualized rates of all-cause death (8.1% vs. 2.5%) and CV death (2.9% vs. 1.2%) were higher in patients receiving 30 mg than in patients receiving 60 mg. The annualized rates of bleeding events and stroke were higher in patients receiving edoxaban 30 mg. **Conclusions:** The results obtained in clinical practice verified the long-term effectiveness and safety of edoxaban, with low annualized event rates. The differences in outcomes according to edoxaban dose may be attributable to the underlying indication associated with each dose level and to clinical differences between patients.

## 1. Introduction

Atrial fibrillation (AF) is among the most common heart conditions, with an estimated global prevalence of 59.7 million individuals in 2019. Its incidence doubles every few decades, mainly due to population aging [1,2,3]. Studies in the Portuguese population over 40 years of age showed that the prevalence of AF was 2.5% [4], reaching 9.0% in patients over 65 [5]. In Spain, AF affects more than 4% of people over 40, impacting over 1 million people [6,7].

AF has high morbidity, including a risk of stroke, heart failure, and other thromboembolic outcomes like cerebral damage [3]. In addition, patients with AF can present with other comorbidities, such as atrial cardiomyopathy (which corresponds to a combination of structural, electrical, or functional changes in the atria), that can affect the progression and recurrence of AF, limit the effectiveness of AF therapy, and promote thrombogenesis and other clinical events, such as the development of heart failure [3,8]. All these conditions contribute to mortality and poor quality of life in patients with AF [3].

For decades, vitamin K antagonists (VKAs), like warfarin, were used to prevent thromboembolic events in AF. However, their risk of intracranial and other major hemorrhages, limited use caused by multiple drug interactions and a narrow therapeutic window, led to the development of non-VKA oral anticoagulants (NOACs), now established as first-line therapy in AF and allowing more widespread prescription [3].

Edoxaban is an NOAC that selectively and reversibly inhibits factor Xa. It is indicated for the prevention of stroke and systemic embolism in non-valvular AF [9]. The European Medicines Agency (EMA) approved its use in 2015, supported by studies such as ENGAGE AF-TIMI 48 [10]. The Edoxaban Treatment in routiNe clinical prActice for patients with non-valvular Atrial Fibrillation Europe (ETNA-AF-Europe) study (Clinicaltrials.gov: NCT02944019) was designed as a single-arm prospective observational study whose objective was to collect real-world data on the safety and effectiveness of edoxaban in patients with AF receiving routine care in Europe [10].

The results of the ETNA-AF-Europe study for the general European population [11,12,13] and 1 year of follow-up in the Spanish population have already been published [14]. The present analysis aims to present the results of the long-term safety and effectiveness of edoxaban observed in the 4-year follow-up in Spain and Portugal (also referred to as Iberia) within the framework of the ETNA-AF-Europe study.

## 2. Materials and Methods

### 2.1. Study Design

The ETNA-AF-Europe study design has already been published [10]. In summary, this was a multinational, multicenter, single-arm, prospective, post-authorization observational study conducted at 776 sites across 10 European countries (Austria, Belgium, Germany, Ireland, Italy, the Netherlands, Portugal, Spain, Switzerland, and the United Kingdom) [13]. In Spain and Portugal, 89 and 9 sites were involved, respectively. This study was approved by the required Competent Authorities and Ethics Committees in all the countries involved.

The study was conducted in Iberia between 2017 and 2022. After inclusion, participants initiated a 48-month follow-up period. There were 4 follow-up checkpoints, one every 12 ± 2 months from baseline. Patients who discontinued edoxaban were monitored annually for an additional 2 years or until the study’s completion, whichever came first [10].

### 2.2. Population

ETNA-AF-Europe’s inclusion and exclusion criteria were broad to collect real-world data. Regarding treatment with edoxaban, two doses could be used: 30 or 60 mg, once daily. The 30 mg dose is indicated for patients with moderate/severe renal impairment (creatinine clearance 15–50 mL/min), low body weight (≤60 kg), or concomitant use of strong P-glycoprotein inhibitors [9,10]. All patients signed the informed consent form before enrollment.

### 2.3. Objectives and Outcome Measures

The primary objective was to assess the real-world safety of edoxaban in AF by evaluating bleeding events (including intracranial hemorrhage (ICH)), drug-related adverse events, and cardiovascular and all-cause mortality.

The secondary objective was to assess the effect of edoxaban on subject-relevant outcomes such as stroke (ischemic and hemorrhagic), systemic embolic events (SEEs), transient ischemic attack (TIA), major adverse cardiovascular events (MACE; a composite endpoint of nonfatal myocardial infarction, nonfatal stroke, nonfatal SEE, and death due to cardiovascular causes or bleeding), venous thromboembolism (VTE) episodes, acute coronary syndromes (ACS), hospitalizations related to cardiovascular conditions, and exposure to edoxaban therapy [10]. Definitions of both any stroke and ICH included hemorrhagic stroke events. Thus, these events were counted as part of both outcomes.

### 2.4. Patient Groups Reported in This Analysis

For this analysis, the two doses of edoxaban that patients were receiving at baseline as part of their regular treatment were considered: 60 or 30 mg.

It was also considered whether these doses were in line with the Summary of Product Characteristics (SmPC) recommendations. Hence, the following groups were established: patients with an SmPC recommendation for 60 mg (60 mg recommended dose or 30 mg non-recommended dose), patients with an SmPC recommendation for 30 mg (30 mg recommended dose or 60 mg non-recommended dose), and patients for whom the dosage rationale could not be confirmed (60 mg or 30 mg unjudgeable dose).

On the other hand, the study results were analyzed according to participants’ gender (male or female). In this article, gender refers to socially constructed roles, behaviors, and identities that take place in a historical and cultural context and which may vary across societies and over time.

In addition, the age of the participants at baseline was also taken into consideration, and patients were categorized into the following groups: <65 years, between 65 and 75 years, and ≥75 years.

Moreover, the results were stratified according to renal function at baseline, establishing the following groups according to participants’ baseline creatinine clearance (CrCl) values: ≤50 mL/min, between 50 and 80 mL/min, and >80 mL/min.

Lastly, the derived CHA2DS2-VASc score at baseline, which estimates the risk of stroke, was used. Patients were categorized as being at low (0–1 points), moderate (2–4 points), or high (>4 points) risk.

### 2.5. Statistical Analysis

Baseline characteristics are reported descriptively as absolute frequencies (n, %) or median and interquartile range (IQR). Effectiveness and safety results are described as annualized event rates (%/year) for the total population and for the previously mentioned subgroups. These rates were calculated as 100% multiplied by the number of patients with at least one event within four years from baseline, divided by the total sum of individual patient observation time (in years). Observation time was measured from the start date until the first event, or, if no event occurred, until the earliest of the following: premature study discontinuation, death, or the last available follow-up date. Patients with missing data were not included in the percentage calculations. When applicable, Kaplan–Meier analysis was performed to illustrate risk over time. Results were reported for the full analysis set (FAS), except for adverse drug reaction (ADR) details, patient status at study conclusion (number of patients who reached the regular end of the trial, time from baseline to premature termination, and reason for premature termination), and distribution of patients based on initial edoxaban dose stratified by SmPC data, which were reported for the baseline analysis set (BAS). Data was analyzed using SAS software (SAS Institute Inc., Cary, NC, USA), version 9.4 or higher. In addition, survival analyses were performed. Cox regression analysis (unadjusted as well as adjusted according to CHA2DS2-VASc score (derived) at baseline) was used to compare the 30 mg and 60 mg doses of edoxaban. No adjustment for multiple testing was made; therefore, all results should be interpreted in a purely descriptive and exploratory way.

### 2.6. Statement on the Use of Artificial Intelligence

No artificial intelligence tools were used for the design or preparation of this study.

## 3. Results

### 3.1. Baseline Characteristics

#### 3.1.1. Total Population and Patients Receiving 60 or 30 mg at Baseline

**Figure 1:**
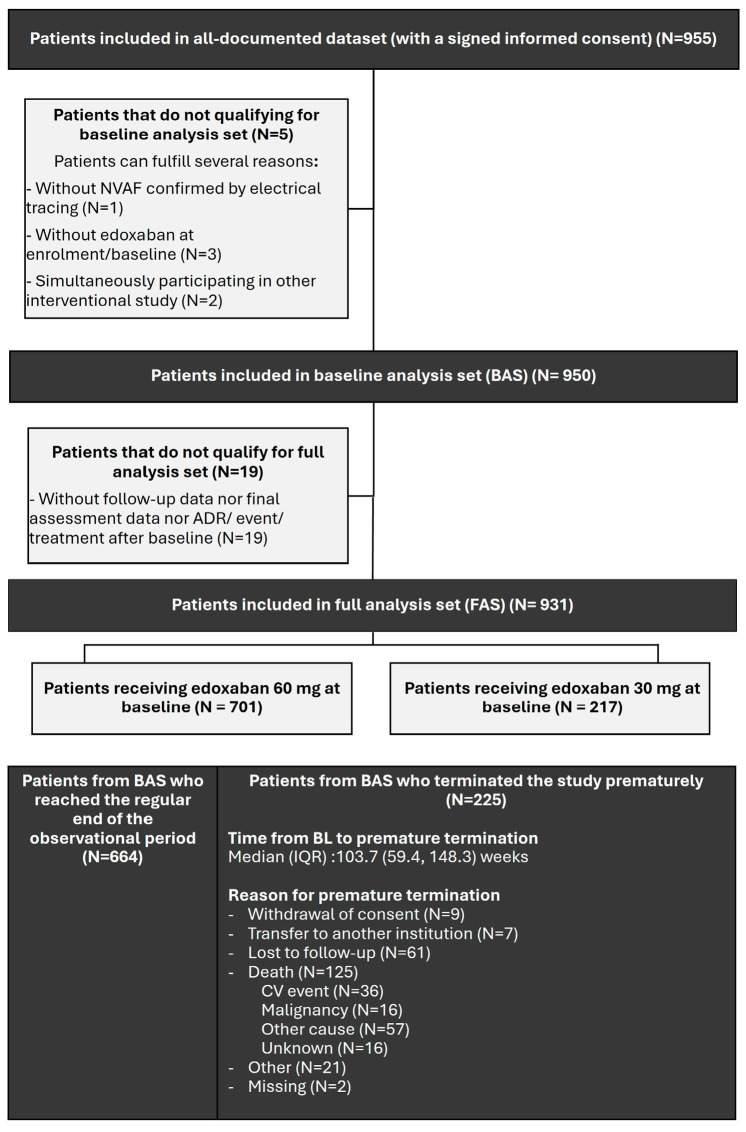
Flow diagram of patients enrolled in Spain and Portugal (FAS).

In Spain and Portugal, 955 patients signed the informed consent form (Figure 1); 950 were included in the BAS and 931 in the FAS (823 from Spain and 108 from Portugal). The baseline characteristics of the participants are presented in Table 1. At baseline, 75.3% of patients were receiving 60 mg edoxaban, and 23.3% were receiving 30 mg. Participants receiving 30 mg were older, had lower body weight, a higher CHA2DS2-VASc score, were more likely to be frail, and had a higher prevalence of most comorbidities compared to those receiving 60 mg.

Regarding participation duration, 664 patients (74.7%) completed follow-up, whereas 225 (25.3%) terminated the study prematurely (Figure 1). The median time from baseline to premature study termination was 103.7 weeks (IQR: 59.4, 148.3). The reasons for premature termination are shown in Figure 1.

Regarding treatment with edoxaban, at the 4-year follow-up (48 months), 88.8% (N = 365) of participants who had available documentation and were still participating in the study were still receiving edoxaban, whereas 11.2% (N = 46) had discontinued treatment. This information was missing for 520 participants, which can be explained by several reasons. First, 225 participants terminated the study prematurely. Consequently, no information was available at the 4-year follow-up. Furthermore, some participants completed the study according to the schedule, but before reaching the full 48 months (due to the predefined visit window of ±2 months). For these participants, no information about their treatment status at 48 months was available. Lastly, some patients were still participating in the study at 48 months but lacked sufficient documentation to determine treatment status.

The median edoxaban exposure was 208.43 weeks. Discontinuations were mostly due to unknown reasons (59.5%), ADRs or clinical events (22.5%), or changes in renal function (4.6%). Among the 178 patients who permanently discontinued treatment with edoxaban, 29.8% (N = 53) started treatment with another NOAC, whereas 5.6% (N = 10) were treated with VKAs.

#### 3.1.2. Patients Receiving 60 or 30 mg in Line with or Not in Line with the SmPC Recommendation at Baseline

The distribution of patients according to baseline edoxaban dose (60 or 30 mg) in line or not in line with SmPC recommendations is described in Table S1.

In Table S2, the baseline characteristics for these groups are included. Most participants (57.7%) received 60 mg, as recommended in the SmPC. Patients receiving the 30 mg recommended dose had the lowest body weight and were more likely to be frail compared to the other groups.

#### 3.1.3. Baseline Characteristics by Gender

Baseline data by gender is shown in Table S3. In the study, 54.9% of patients were males. Females were older, had lower body weight, and were more likely to be frail, whereas males had a higher prevalence of most CV comorbidities.

### 3.2. Clinical Outcomes Following 4-Year Follow-Up in Spain and Portugal

#### 3.2.1. Total Population and Patients Receiving 60 or 30 mg Dose at Baseline

Regarding the primary outcomes, the annualized rates of all-cause (8.1% vs. 2.5%) and CV death (2.9% vs. 1.2%), any bleeding events (6.2% vs. 3.6%), major bleeding events (3.2% vs. 1.1%), gastrointestinal bleeding events (3.2% vs. 1.3%), and major gastrointestinal bleeding events (1.5% vs. 0.6%) were higher in the 30 mg edoxaban group than in the 60 mg group. There were only five ICH events in total (0.2) (Table 2).

Regarding the secondary outcomes, annualized event rates of stroke, TIA, MACE, and hospitalizations related to cardiovascular conditions were higher in patients receiving 30 mg (Table 2).

There were 12 ADRs during the 4-year follow-up, 10 of which were related to the treatment (Table 3). Beyond AF-related events, the ADRs reported included nausea, pain, epistaxis, pruritus, and rash.

In addition, survival analyses were performed. The results of the Cox regression analysis for time to first occurrence of all-cause death, CV death, major bleeding events, ICH, stroke, ischemic stroke, and hemorrhagic stroke are shown in Table 4. All results show a higher risk for the 30 mg group.

**Figure 2:**
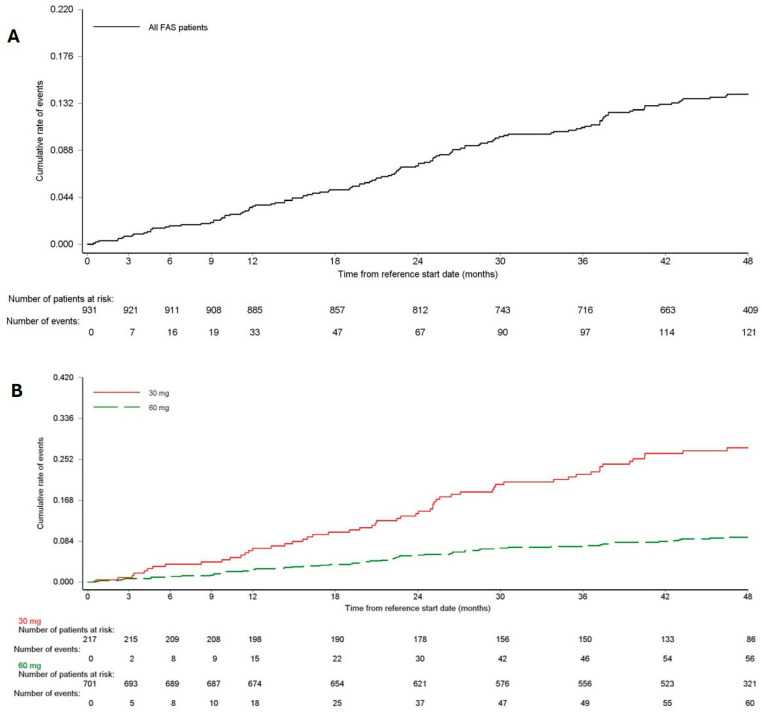
Kaplan–Meier plots for the time to death from any cause for the total population and by edoxaban dose at baseline (FAS). (**A**) Total population. (**B**) By initial edoxaban dose. FAS, full analysis set.

**Figure 3:**
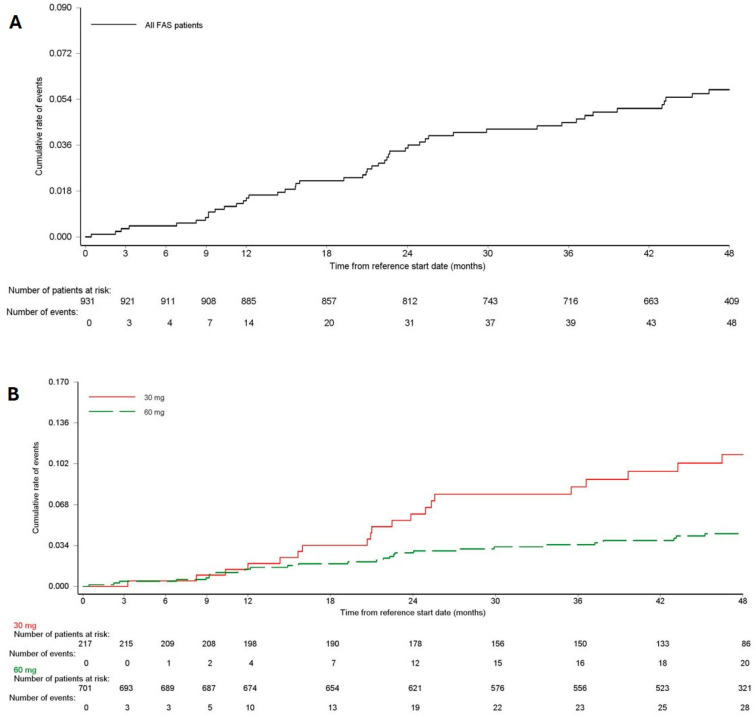
Kaplan–Meier plots for the time to cardiovascular death for the total population and by edoxaban dose at baseline (FAS). (**A**) Total population. (**B**) By initial edoxaban dose. FAS, full analysis set.

**Figure 4:**
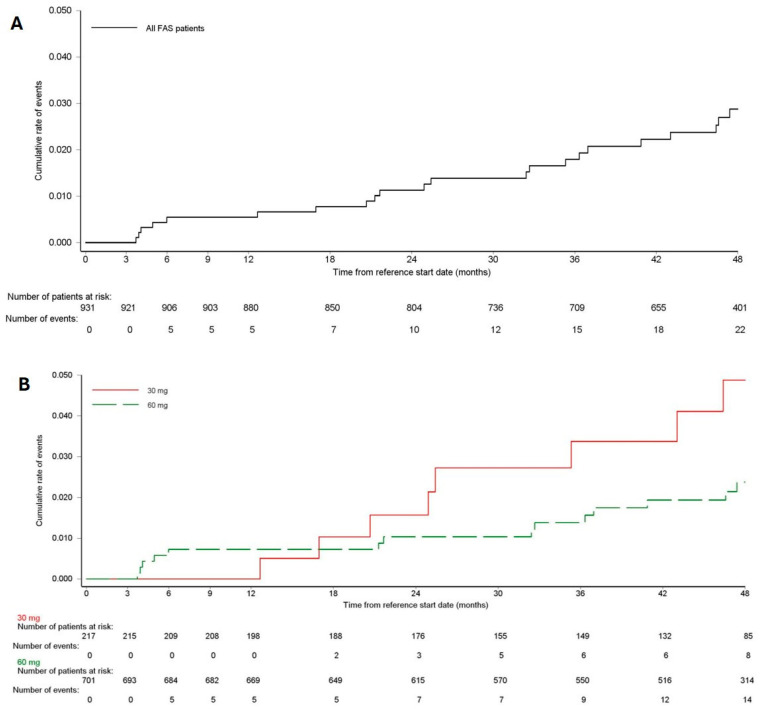
Kaplan–Meier plots for the time to first occurrence of stroke for the total population and by edoxaban dose at baseline (FAS). (**A**) Total population. (**B**) By initial edoxaban dose. FAS, full analysis set.

**Figure 5:**
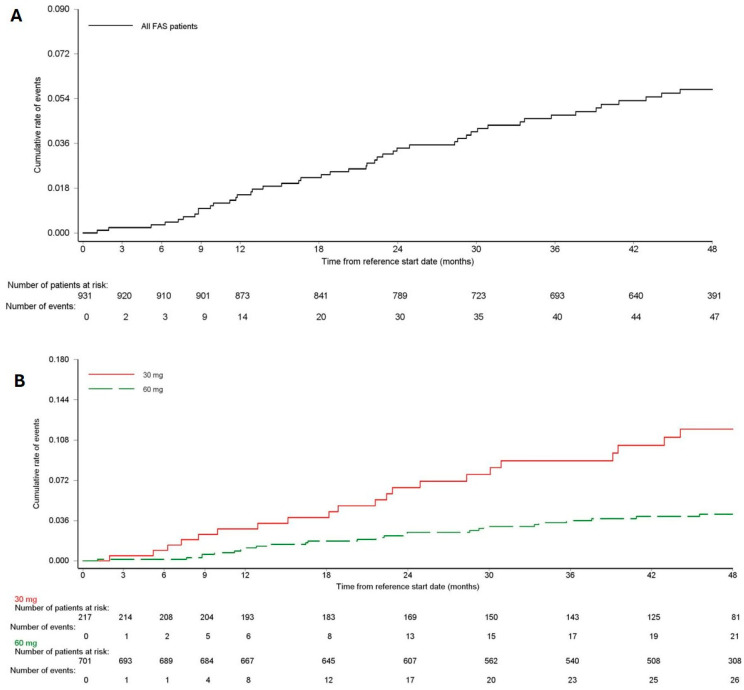
Kaplan–Meier plots for the time to first major bleeding event for the total population and by edoxaban dose at baseline (FAS). (**A**) Total population. (**B**) By initial edoxaban dose. FAS, full analysis set.

**Figure 6:**
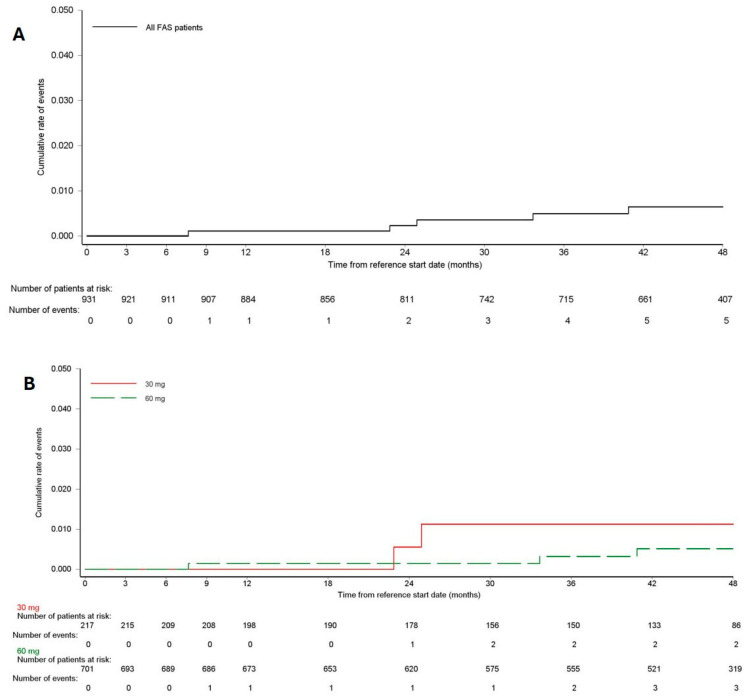
Kaplan–Meier plots for the time to first occurrence of ICH for the total population and by edoxaban dose at baseline (FAS). (**A**) Total population. (**B**) By initial edoxaban dose. FAS, full analysis set.

Kaplan–Meier plots (Figure 2, Figure 3, Figure 4, Figure 5 and Figure 6) illustrate the time to first occurrence of death, CV death, stroke, major bleeding event, and ICH, showing a nearly linear trend over the 4 years.

**Figure 7:**
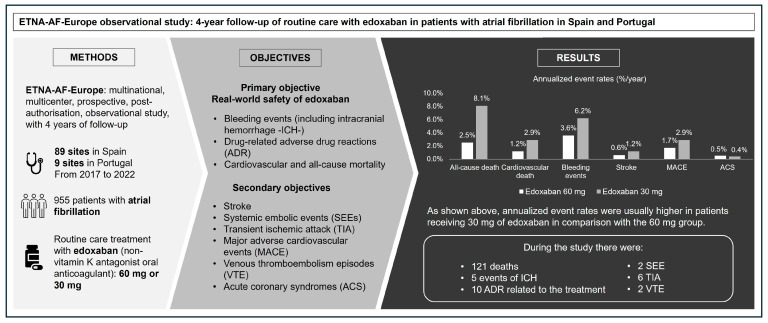
Design of ETNA-AF-Europe study and results of the 4-year follow-up in Spain and Portugal.

A summary of this study and its results is shown in Figure 7.

#### 3.2.2. Patients Receiving 60 or 30 mg in Line with or Not in Line with the SmPC Recommendation

The annualized event rates of all-cause death and CV death were highest in patients receiving the 30 mg recommended edoxaban dose (Table 5). Regarding the bleeding outcomes, the 60 mg non-recommended dose group had the highest rate of any bleeding events, the 30 mg recommended dose group had the highest rate of major bleeding events, and the 30 mg non-recommended dose group had the highest rates of gastrointestinal bleeding events and major gastrointestinal bleeding events (Table 5). Regarding the secondary outcomes, the 60 mg non-recommended dose group had slightly higher annualized event rates of stroke (Table 5).

#### 3.2.3. Patients Stratified by Gender

There were slightly higher annualized rates of all-cause death in males and CV death in females (Table 6), without a relevant difference. Except for the annualized rate for any bleeding events, which was higher in males, all rates of bleeding events were slightly higher in females (Table 6). Also, females had slightly higher rates of stroke.

Figures S1–S5 show Kaplan–Meier plots for death, CV death, stroke, major bleeding event, and ICH in both genders.

#### 3.2.4. Patients Stratified by Age Group at Baseline

When evaluating primary outcomes by age group (Table 7), the annualized rates of all-cause death (1.6% for patients < 65 years; 1.3% for patients between 65 and 75 years; and 6.5% for patients ≥ 75 years), CV death (0.7%, 0.4%, and 2.6%, respectively) were higher in participants aged ≥75 years compared to the other groups. Regarding bleeding events, this group also had higher annualized rates of any bleeding events (1.9%, 2.7%, and 6.0%), major bleeding events (0.4%, 0.7%, and 2.5%), gastrointestinal bleeding events (0.7%, 0.9%, and 2.7%), and major gastrointestinal bleeding events (0.2%, 0.3%, and 1.3%) compared to the other groups.

Regarding the secondary outcomes, participants aged ≥75 years had the highest annualized event rates of stroke, MACE, and hospitalizations related to cardiovascular conditions.

Figures S6–S10 show Kaplan–Meier plots for death, CV death, stroke, major bleeding event, and ICH in all age groups.

#### 3.2.5. Patients Stratified by Renal Function at Baseline

Table 8 shows the results of the study, considering the renal function of participants at baseline. When evaluating the primary outcomes, the group of patients with the lowest CrCl (≤50 mL/min) had higher annualized rates of all-cause death (8.5%, 4.4%, and 1.6%), CV death (3.4%, 1.7%, and 0.7%), any bleeding events (7.7%, 4.4%, and 3.1%), major bleeding events (3.7%, 1.9%, and 0.5%), and gastrointestinal bleeding events (2.8%, 2.2%, and 1.1%) than the other groups (≤50 mL/min, between 50 and 80 mL/min, and >80 mL/min, respectively). However, for major gastrointestinal bleeding events, the annualized rates were the same in the ≤50 mL/min group and the group with CrCl between 50 and 80 mL/min.

The results of the secondary outcomes show that participants with CrCl ≤ 50 mL/min had the highest annualized rates of stroke, TIA, MACE, and hospitalizations related to cardiovascular conditions.

Figures S11–S15 show Kaplan–Meier plots for death, CV death, stroke, major bleeding event, and ICH in all groups by renal function.

#### 3.2.6. Patients Stratified by CHA2DS2-VASc Risk Group at Baseline

Lastly, the CHA2DS2-VASc score of participants at baseline was also considered (Table 9). Patients were classified into three categories based on their CHA2DS2-VASc score at baseline: low risk (0–1 pt), moderate risk (2–4 pts), and high risk (>4 pts). The annualized rates of all-cause death (1.1% for low risk; 3.5% for moderate risk; and 6.3% for high risk), CV death (0.7%, 1.3%, and 2.8%), any bleeding events (1.1%, 4.1%, and 5.9%), major bleeding events (0.0%, 1.5%, 2.4%), gastrointestinal bleeding events (0.0%, 1.6%, and 3.1%), and major gastrointestinal bleeding events (0.0%, 0.6%, and 2.0%) were higher in patients with a CHA2DS2-VASc score >4 pts at baseline compared with the other groups.

Regarding the secondary outcomes, the annualized rates of stroke, MACE, and hospitalizations related to cardiovascular conditions were also higher in the CHA2DS2-VASc high-risk group.

Figures S16–S20 show Kaplan–Meier plots for death, CV death, stroke, major bleeding event, and ICH in all groups by CHA2DS2-VASc score.

## 4. Discussion

The ETNA-AF-Europe study results in Spain and Portugal corroborate the safety and effectiveness of edoxaban in standard AF clinical practice over a 4-year follow-up period. These findings confirm the information obtained on the general European population in the ETNA-AF-Europe study at 1 [11], 2 [12], and 4 years of follow-up [13], and at 1 year in the Spanish population [14]. Edoxaban’s benefits in AF have previously been confirmed in clinical trials, such as the ENGAGE AF–TIMI 48 study [15], and are supported by meta-analyses of clinical trials and observational studies [16,17,18]. Alongside prospective observational studies with other NOACs showing satisfactory results [19,20,21,22,23], evidence supports the real-world effectiveness and safety of NOACs in AF.

It should be noted that this trial focused on evaluating the safety and effectiveness of the two doses of edoxaban approved for stroke prevention in AF patients, the 60 and 30 mg doses. Although a 15 mg dose has been evaluated for the prevention of stroke in elderly patients with AF who are not candidates for oral anticoagulant therapy (ELDERCARE-AF trial), this study was conducted solely in a Japanese population; consequently, this use is not authorized in the SmPC in Europe, and the 15 mg dose is intended solely for transitioning patients from the 30 mg dose to a VKA [9,24]. After the recommended International Normalized Ratio (INR) is achieved, edoxaban 15 mg should be discontinued. For this reason, patients receiving the off-label 15 mg dose were not included in this study.

Regarding the primary outcomes evaluated in this study, the annualized rates of all-cause death with edoxaban were lower than those reported in the literature for warfarin [25,26,27]. For major bleeding events, the annualized rates were also lower compared with the results for warfarin from previous studies [15,25,26,27,28,29]. For ICH events, the annualized rates with edoxaban were also lower than those reported for warfarin [28,29,30,31]. The rates of death, major bleeding events, and ICH were in line with a recent meta-analysis reporting a low incidence of these events with edoxaban [16].

Regarding the secondary outcomes, the present results showed that the annualized stroke rates with edoxaban were also lower than those reported for warfarin [15,25,26,27,30] and were consistent with previous real-world studies reporting a low incidence of stroke [16].

### 4.1. Results for the Total Population and Patients Receiving 60 or 30 mg at Baseline Compared with the ETNA-AF-Europe Study General Results

The annualized rates of all-cause death in Spain and Portugal (3.8% total; 2.5% for 60 mg; and 8.1% for 30 mg) were similar, although slightly lower than those reported in the general European analysis of the ETNA-AF-Europe study (4.1%, 2.8%, and 8.4%, respectively) [13]. The annualized rates of CV death (1.5%, 1.2%, and 2.9%) in Iberia were also similar but slightly higher than those in the general European population (1.0%, 0.7%, and 2.0%) [13]. The results for the total population and by edoxaban dose showed that edoxaban effectiveness in Iberia followed the same pattern as in the general European population, and death rates were higher in patients receiving 30 mg compared with 60 mg.

When comparing bleeding outcomes in the Iberian population with those in the same groups of the general European cohort, it can be observed that Portuguese and Spanish patients had slightly higher annualized rates of any bleeding events (4.1%, 3.6%, and 6.2% vs. 2.9%, 2.8%, and 3.4%), major bleeding events (1.5%, 1.1%, and 3.2% vs. 0.8%, 0.7%, and 1.2%), gastrointestinal bleeding events (1.7%, 1.3%, and 3.2% vs. 1.0%. 0.9%, and 1.4%), and major gastrointestinal bleeding events (0.8%, 0.6%, and 1.5% vs. 0.4%, 0.3%, and 0.6%) than the European cohort [13]. In both populations, these rates were higher in the 30 mg edoxaban group compared to the 60 mg group. This could be due to chance, patient characteristics, or underlying bleeding risk factors.

The annualized stroke rates (0.7%, 0.6%, and 1.2% vs. 0.6%, 0.6%, and 0.9%) were very similar between the Iberian and general European populations, and in both cases, the rates were slightly higher in patients receiving 30 mg of edoxaban at baseline [13]. This also shows the effectiveness of edoxaban in preventing these events in patients with AF.

In general, as shown above, participants receiving edoxaban 30 mg usually had higher rates of the mentioned events. It should be noted that these patients were older, more likely to be frail, and had a higher prevalence of comorbidities at baseline, which may be related to the observed results. Additionally, the 30 mg dose is usually indicated for patients with specific clinical factors, such as moderate or severe renal impairment or concomitant use of strong P-glycoprotein inhibitors. Hence, the differences may also be attributable to the underlying indication associated with each dose level. These results support that bleeding risk depends on patient risk profile and align with the European analysis.

### 4.2. Results Stratified by Patients Receiving 60 or 30 mg of Edoxaban in Line with or Not in Line with the SmPC Recommendation Compared with the ETNA-AF-Europe Study General Results

In this study, the results (Table S1) showed that in Iberia, “overdosing” (use of 60 mg edoxaban, although a reduction to 30 mg was recommended by the SmPC, 8.5%) was more common than “underdosing” and treating with the 30 mg dose without valid reasons (4.7%). The prevalence of underdosing was lower than in the general European analysis (6.2%) and much lower than in previous studies and meta-analyses [32,33,34]. NOACs are frequently underdosed, especially in old and frail patients, to minimize bleeding risk; however, this can increase mortality [19,32,33,34]. Overdosing also carries risks, since it may be associated with poor outcomes such as higher risk of thromboembolism, all-cause death, and major bleeding [32,35]. Hence, following SmPC dosing recommendations is crucial to avoid a lack of efficacy or the occurrence of undesirable events.

The results for patients receiving edoxaban in line with or not in line with the SmPC were also evaluated. The annualized rates of death, CV death, and major bleeding events were highest in patients receiving the 30 mg recommended dose, both in Iberia and in the general European population [13]. Although in the Iberian population, the 60 mg non-recommended dose group had slightly higher annualized rates of stroke than the other groups, in the European cohort, this rate was slightly higher in the 30 mg recommended dose group.

Hence, overall, patients with an SmPC recommendation for 30 mg (30 mg recommended dose and 60 mg non-recommended dose) usually had the highest event rates both in the Iberian population and in the general European analysis. In Spain and Portugal, patients receiving the 30 mg recommended dose were more likely to be frail, and they received this dose according to the SmPC because they presented other specific clinical factors, as mentioned previously, that are associated with the indication of each dose level and that may have influenced the results obtained.

### 4.3. Results Stratified by Gender

According to the edoxaban SmPC, no dose adjustment is required based on gender, and in its pivotal clinical trials (such as ENGAGE AF-TIMI 48), the efficacy results by gender were consistent with those of the overall population [9,36].

In this study, females were older and more likely to be frail, while males had a higher prevalence of most CV comorbidities. However, the analysis showed that although differences were observed in the annualized event rates for the primary and secondary outcomes, these differences were generally minimal.

### 4.4. Results Stratified by Age, Renal Function, and CHA2DS2-VASc Risk Group at Baseline

The results of this study show that the participants aged ≥75 years, patients with CrCl ≤50 mL/min, and patients at high risk (>4 pts) on the CHA2DS2-VASc score had higher annualized rates in most of the principal events analyzed, but they were also more likely to have relevant concomitant comorbidities and a more vulnerable profile. It should be noted that, as indicated in the edoxaban SmPC, in participants with a moderate or severe renal impairment (CrCl 15–50 mL/min), a 30 mg edoxaban dose is recommended, since this condition can affect edoxaban pharmacokinetics [9].

It was generally observed for certain outcomes that as age or CHA2DS2-VASc scores increased or CrCl values decreased, the annualized rates of these events rose. It can be hypothesized that this is related to the increased frailty in these patients, their predisposition to such events, and the presence of other concomitant conditions.

However, it should be noted that edoxaban is indicated in patients with NVAF with one or more risk factors, including age ≥75 years, since these high-risk patients benefit from edoxaban in the prevention of stroke and systemic embolism [9,36]. In addition, it has been previously shown that age does not have an additional clinically significant effect on edoxaban pharmacokinetics [9,36]. These results therefore reinforce the need for careful clinical assessment and individualized management in elderly patients, while supporting the continued use of edoxaban as an effective preventive strategy in this high-risk group.

Regarding renal function, the progressive increase in event rates observed with declining CrCl reinforces the importance of careful dose selection and close clinical monitoring in patients with impaired renal function. Ultimately, renal dysfunction appears not only as a pharmacokinetic determinant of edoxaban dosing but also as a marker of overall vulnerability, helping to explain the higher burden of events seen in this subgroup.

Lastly, the progressive rise in event rates as the CHA2DS2-VASc scores increase reinforces the value of the CHA2DS2-VASc score as a practical and informative tool for stratifying patient risk. Ultimately, these results support the importance of integrating structured risk assessment instruments into routine clinical decision-making to guide individualized treatment strategies and optimize patient outcomes.

### 4.5. Limitations

Since this was an open-label observational study with no control group, there are certain limitations associated with the study design. First, data collection was limited to the information available in routine clinical practice. In addition, study visits were spaced 12 months apart, which may have favored a recall bias. Second, since there was no control arm, direct comparisons with other medications could not be made. Third, the study was not blinded, so bias due to patient and investigator awareness of the treatment under study cannot be excluded. To compensate for potential differences in baseline characteristics, the results from an adjusted Cox regression with the CHA2DS2-VASc score at baseline as an additional covariate were presented. However, although the CHA2DS2-VASc score is a good surrogate for potential differences, it may not capture all relevant factors. Hence, as no statistical adjustment was applied to account for all baseline differences between dose groups, and as these dosage ranges apply to populations with different characteristics, the conclusions remain descriptive. Lastly, due to the long duration of follow-up, the loss of patients during the study must also be taken into account, since 225 patients (25.3%) discontinued the study prematurely. This discontinuity means that some data are unavailable, which may affect the results, since no sensitivity analysis was performed. This loss of data is significant, for example, for the status of the treatment with edoxaban at the end of follow-up (48 months), which is missing for 520 participants. It should be noted that the analyses were conducted according to the initial dose of edoxaban used by patients, without taking into account the final dose used or whether treatment was discontinued, which may also limit this study’s conclusions.

## 5. Conclusions

This 4-year observational study of edoxaban treatment for AF in clinical practice in Spain and Portugal confirms its effectiveness and safety. The annualized rates of death, major bleeding, ICH, and stroke were low, consistent with the information already reported in the literature on edoxaban. It should be noted that the annualized event rates were higher in patients receiving edoxaban 30 mg compared to those receiving 60 mg and that the 30 mg group had several risk factors (i.e., older, more likely to be frail, a higher prevalence of CV comorbidities), which probably influenced these outcomes. These differences may also be attributable to the underlying indication associated with each dose level, since the 30 mg dose is usually indicated for patients with specific clinical factors such as moderate or severe renal impairment or concomitant use of strong P-glycoprotein inhibitors. These results also align with the analysis of the general European population in the ETNA-AF-Europe study.

In conclusion, this study supports the real-world effectiveness and safety of long-term edoxaban treatment in routine AF care across Spain and Portugal, which had previously been demonstrated in clinical trials and in the overall results of the ETNA-AF-Europe study.

## Figures and Tables

**Table 1:** Baseline characteristics for the total population and by edoxaban dose at baseline (FAS).

Characteristic, N (%) or Median (IQR) *	Total (*n* = 931)	Edoxaban 60 mg (*n* = 701)	Edoxaban 30 mg (*n* = 217)
Age, years	75.0 (68.0, 81.0)	72.0 (66.0, 79.0)	81.0 (75.0, 86.0)
Male	511 (54.9)	410 (58.5)	92 (42.4)
Weight, kg	77.0 (67.0, 85.0)	79.0 (70.0, 87.0)	67.5 (57.0, 81.0)
Body mass index, kg/m^2^	28.0 (25.4, 31.2)	28.5 (26.0, 31.3)	26.2 (23.2, 30.8)
Overweight (≥25 and <30 kg/m^2^)	365 (43.6)	291 (46.6)	67 (33.2)
Obese (≥30 kg/m^2^)	290 (34.6)	229 (36.7)	59 (29.2)
CrCl [mL/min]	70.11 (52.73, 89.21)	76.85 (60.60, 95.10)	45.09 (36.42, 59.07)
<15	0	0	0
≥15–≤30	23 (2.8)	2 (0.3)	20 (9.9)
>30–≤50	161 (19.3)	53 (8.5)	106 (52.2)
>50–≤80	341 (40.9)	272 (43.9)	63 (31.0)
>80	309 (37.1)	293 (47.3)	14 (6.9)
History of renal impairment	526 (57.4)	327 (47.3)	190 (88.8)
SBP, mmHg	130.0 (120.0, 142.0)	130.0 (120.0, 140.0)	131.5 (120.0, 145.0)
Uncontrolled hypertension episode (SBP >160 mmHg)	101 (12.8)	70 (11.8)	27 (14.8)
CHA2DS2-VASc risk score	3.0 (2.0, 4.0)	3.0 (2.0, 4.0)	4.0 (3.0, 5.0)
Low (0–1 pt)	83 (9.1)	78 (11.3)	4 (1.9)
Moderate (2–4 pts)	657 (71.7)	495 (71.8)	152 (71.0)
High (>4 pts)	176 (19.2)	116 (16.8)	58 (27.1)
Modified HAS-BLED score	2.0 (1.0, 2.0)	2.0 (1.0, 2.0)	2.0 (2.0, 3.0)
Low (0–1 pt)	270 (35.1)	247 (42.7)	21 (11.7)
Medium (2 pts)	355 (46.1)	239 (41.3)	109 (60.9)
High (>2 pts)	145 (18.8)	93 (16.1)	49 (27.4)
Current smoker	42 (5.1)	38 (6.1)	4 (2.0)
Frailty (investigator-assessed)	107 (11.9)	50 (7.4)	55 (26.7)
Chronic hepatic disease	16 (1.8)	12 (1.8)	3 (1.4)
Type of NVAF			
Paroxysmal	447 (48.0)	351 (79.2)	91 (20.5)
Persistent	196 (21.0)	153 (80.1)	38 (19.9)
Long-standing persistent or permanent	288 (30.9)	196 (69.0)	88 (31.0)
History of stroke	87 (9.3)	70 (10.0)	17 (7.8)
Hemorrhagic stroke	4 (0.4)	2 (0.3)	2 (0.9)
Ischemic stroke	79 (8.5)	65 (9.3)	14 (6.5)
Unknown stroke type	4 (0.4)	3 (0.4)	1 (0.5)
History of TIA (transient ischemic stroke)	35 (3.8)	24 (3.4)	10 (4.6)
History of CV disease			
Hypertension	710 (76.3)	528 (75.3)	171 (78.8)
Coronary heart disease	103 (11.1)	68 (9.8)	34 (15.8)
Peripheral artery disease	21 (2.3)	15 (2.2)	6 (2.8)
Valvular heart disease	105 (11.4)	68 (9.8)	35 (16.2)
History of DM	247 (26.7)	187 (26.9)	57 (26.3)
History of COPD	73 (7.9)	58 (8.3)	12 (5.6)

* Percentages are based on the total number of patients with non-missing observations (excluding patients with missing or unknown data). COPD, chronic obstructive pulmonary disease; CrCl, creatinine clearance; CV, cardiovascular; DM, diabetes mellitus; HAS-BLED, hypertension, abnormal liver/renal function, stroke history, bleeding history or predisposition, labile INR, elderly, drug/alcohol usage; IQR, interquartile range; NVAF, non-valvular atrial fibrillation; SBP, systolic blood pressure; TIA, transient ischemic stroke.

**Table 2:** Clinical outcomes for the total population and by edoxaban dose at baseline (FAS).

Relevant Clinical Event Annualized Event Rates N (%/Year) [95% CI] *	Total (*n* = 931)	60 mg (*n* = 701)	30 mg (*n* = 217)
All-cause death	121 (3.8) [3.14;4.50]	60 (2.5) [1.84;3.09]	56 (8.1) [5.98;10.22]
CV death	48 (1.5) [1.09;1.94]	28 (1.2) [0.72;1.58]	20 (2.9) [1.63;4.16]
Any bleeding events	120 (4.1) [3.35;4.82]	81 (3.6) [2.80;4.36]	39 (6.2) [4.25;8.15]
Major bleeding events	47 (1.5) [1.08;1.95]	26 (1.1) [0.67;1.50]	21 (3.2) [1.80;4.50]
ICH events	5 (0.2) [0.02;0.30]	3 (0.1) [0.00;0.26]	2 (0.3) [0.00;0.69]
Gastrointestinal bleeding events	53 (1.7) [1.26;2.19]	32 (1.3) [0.88;1.82]	21 (3.2) [1.83;4.56]
Major gastrointestinal bleeding events	24 (0.8) [0.46;1.07]	14 (0.6) [0.28;0.88]	10 (1.5) [0.56;2.39]
Stroke	22 (0.7) [0.41;0.99]	14 (0.6) [0.28;0.88]	8 (1.2) [0.36;1.97]
Ischemic stroke	19 (0.6) [0.33;0.88]	12 (0.5) [0.22;0.78]	7 (1.0) [0.26;1.77]
Hemorrhagic stroke	2 (0.1) [0.00;0.15]	1 (<0.1) [0.00;0.12]	1 (0.1) [0.00;0.43]
SEEs	2 (0.1) [0.00;0.15]	2 (0.1) [0.00;0.20]	0 (0.0) [0.00;0.00]
TIA	6 (0.2) [0.04;0.34]	2 (0.1) [0.00;0.20]	4 (0.6) [0.01;1.16]
MACE	60 (1.9) [1.44;2.41]	40 (1.7) [1.15;2.19]	20 (2.9) [1.65;4.22]
VTE episodes	2 (0.1) [0.00;0.15]	1 (<0.1) [0.00;0.12]	1 (0.1) [0.00;0.43]
ACS	16 (0.5) [0.26;0.76]	13 (0.5) [0.25;0.83]	3 (0.4) [0.00;0.93]
Hospitalizations related to cardiovascular condition	225 (8.2) [7.16;9.31]	159 (7.5) [6.35;8.69]	62 (10.6) [8.00;13.30]

* Percentages are based on the total number of patients with non-missing observations (excluding patients with missing or unknown data). ACS, acute coronary syndrome; CV, cardiovascular; MACE, major adverse cardiovascular events; SEEs, systemic embolic events; TIA, transient ischemic attack; VTE, venous thromboembolism.

**Table 3:** Adverse drug reactions in the total population and by edoxaban dose at baseline (BAS).

Adverse Drug Reaction Details, N (%)	Total (*n* = 950)	60 mg (*n* = 714)	30 mg (*n* = 221)
Total no. of ADRs *	12	7	5
Total no. of patients with at least one ADR	10 (1.1)	5 (0.7)	5 (2.3)
Total no. of patients with at least one serious ADR	4 (0.4)	3 (0.4)	1 (0.5)
ADRs related to the treatment **	10 (90.9)	5 (83.3)	5 (100)

* ADR, adverse drug reaction. ** This percentage refers to the number of patients with at least one ADR.

**Table 4:** Clinical outcomes. Survival analysis: Cox regression for the time to first occurrence for initial edoxaban dose at baseline (FAS).

Clinical Event	Unadjusted Analysis: HR for 30 mg vs. 60 mg (=Reference Category) [95% CI] *	Adjusted Analysis by CHA2DS2-VASc Score (Derived): HR for 30 mg vs. 60 mg (=Reference Category) [95% CI] *
All-cause death	3.272 [2.269;4.711]	2.955 [2.032;4.293]
CV death	2.499 [1.389;4.414]	2.322 [1.276;4.162]
Major bleeding event	2.894 [1.612;5.135]	2.542 [1.411;4.526]
ICH event	2.367 [0.312;14.288]	2.163 [0.283;13.160]
Stroke	2.029 [0.810;4.738]	1.821 [0.720;4.311]
Ischemic stroke	2.069 [0.770;5.145]	1.878 [0.690;4.745]
Hemorrhagic stroke	3.659 [0.145;92.519]	3.756 [0.148;95.069]

* HR: hazard ratio.

**Table 5:** Clinical outcomes by initial edoxaban dose (60 mg or 30 mg) in line with and not in line with SmPC recommendations (FAS).

Relevant Clinical Event Annualized Event Rates N (%/Year) [95% CI] *	Patients with SmPC Recommendation for 60 mg		Patients with SmPC Recommendation for 30 mg		Patients with No Confirmation on Reason for Choice of Dosing	
	60 mg Recommended Dose (*n* = 530)	30 mg Non-Recommended Dose (*n* = 45)	30 mg Recommended Dose (*n* = 159)	60 mg Non-Recommended Dose (*n* = 79)	60 mg (*n* = 92)	30 mg (*n* = 13)
All-cause death	49 (2.7) [1.93;3.43]	7 (4.3) [1.11;7.43]	48 (9.9) [7.09;12.69]	7 (2.6) [0.68;4.56]	4 (1.2) [0.02;2.35]	1 (2.4) [0.00;7.07]
CV death	22 (1.2) [0.70;1.71]	1 (0.6) [0.00;1.81]	18 (3.7) [2.00;5.42]	5 (1.9) [0.23;3.51]	1 (0.3) [0.00;0.88]	1 (2.4) [0.00;7.07]
Any bleeding events	60 (3.5) [2.64;4.42]	9 (6.2) [2.16;10.30]	28 (6.3) [3.97;8.63]	17 (7.1) [3.74;10.51]	4 (1.2) [0.02;2.44]	2 (5.0) [0.00;11.87]
Major bleeding events	19 (1.1) [0.58;1.53]	3 (1.9) [0.00;3.96]	17 (3.7) [1.92;5.40]	7 (2.7) [0.70;4.72]	0 (0.0) [0.00;0.00]	1 (2.5) [0.00;7.26]
ICH events	1 (0.1) [0.00;0.16]	0 (0.0) [0.00;0.00]	2 (0.4) [0.00;0.98]	2 (0.8) [0.00;1.81]	0 (0.0) [0.00;0.00]	0 (0.0) [0.00;0.00]
Gastrointestinal bleeding events	24 (1.3) [0.81;1.88]	8 (5.4) [1.64;9.06]	13 (2.8) [1.27;4.31]	6 (2.3) [0.46;4.19]	2 (0.6) [0.00;1.45]	0 (0.0) [0.00;0.00]
Major gastrointestinal bleeding events	11 (0.6) [0.25;0.97]	3 (1.9) [0.00;3.96]	7 (1.5) [0.38;2.56]	3 (1.1) [0.00;2.44]	0 (0.0) [0.00;0.00]	0 (0.0) [0.00;0.00]
Stroke	9 (0.5) [0.17;0.82]	1 (0.6) [0.00;1.83]	7 (1.4) [0.37;2.52]	4 (1.5) [0.03;3.00]	1 (0.3) [0.00;0.89]	0 (0.0) [0.00;0.00]
Ischemic stroke	7 (0.4) [0.10;0.67]	1 (0.6) [0.00;1.83]	6 (1.2) [0.25;2.23]	4 (1.5) [0.03;3.00]	1 (0.3) [0.00;0.89]	0 0.0 [0.00;0.00]
Hemorrhagic stroke	1 (0.1) [0.00;0.16]	0 (0.0) [0.00;0.00]	1 (0.2) [0.00;0.61]	0 (0.0) [0.00;0.00]	0 (0.0) [0.00;0.00]	0 (0.0) [0.00;0.00]
SEEs	1 (0.1) [0.00;0.16]	0 (0.0) [0.00;0.00]	0 (0.0) [0.00;0.00]	1 (0.4) [0.00;1.11]	0 (0.0) [0.00;0.00]	0 (0.0) [0.00;0.00]
TIA	2 (0.1) [0.00;0.26]	0 (0.0) [0.00;0.00]	4 (0.8) [0.02;1.65]	0 (0.0) [0.00;0.00]	0 (0.0) [0.00;0.00]	0 (0.0) [0.00;0.00]
MACE	28 (1.6) [0.98;2.13]	1 (0.6) [0.00;1.83]	18 (3.8) [2.03;5.51]	10 (3.9) [1.46;6.24]	2 (0.6) [0.00;1.43]	1 (2.4) [0.00;7.10]
VTE	1 (0.1) [0.00;0.16]	0 (0.0) [0.00;0.00]	0 (0.0) [0.00;0.00]	0 (0.0) [0.00;0.00]	0 (0.0) [0.00;0.00]	1 (2.4) [0.00;7.16]
ACS	9 (0.5) [0.17;0.82]	0 (0.0) [0.00;0.00]	2 (0.4) [0.00;1.00]	4 (1.5) [0.03;3.04]	0 (0.0) [0.00;0.00]	1 (2.4) [0.00;7.10]
Hospitalizations related to cardiovascular conditions	125 (7.9) [6.54;9.33]	12 (8.8) [3.84;13.85]	47 (11.6) [8.27;14.90]	23 (10.3) [6.07;14.45]	11 (3.5) [1.43;5.57]	3 (7.3) [0.00;15.59]

* Percentages are based on the total number of patients with non-missing observations (excluding patients with missing or unknown data). ACS, acute coronary syndrome; CV, cardiovascular; MACE, major adverse cardiovascular events; SEEs, systemic embolic events; TIA, transient ischemic attack; VTE, venous thromboembolism.

**Table 6:** Clinical outcomes by gender (FAS).

Relevant Clinical Event Annualized Event Rates N (%/Year) [95% CI] *	Male (*n* = 511)	Female (*n* = 420)
All-cause death	67 (3.9) [2.98;4.85]	54 (3.7) [2.72;4.70]
CV death	24 (1.4) [0.84;1.96]	24 (1.6) [0.99;2.31]
Any bleeding events	67 (4.2) [3.23;5.26]	53 (3.9) [2.85;4.95]
Major bleeding events	21 (1.2) [0.71;1.78]	26 (1.8) [1.13;2.54]
ICH events	2 (0.1) [0.00;0.28]	3 (0.2) [0.00;0.44]
Gastrointestinal bleeding events	28 (1.7) [1.06;2.32]	25 (1.8) [1.07;2.46]
Major gastrointestinal bleeding events	12 (0.7) [0.31;1.11]	12 (0.8) [0.36;1.31]
Stroke	9 (0.5) [0.18;0.87]	13 (0.9) [0.41;1.39]
Ischemic stroke	7 (0.4) [0.11;0.71]	12 (0.8) [0.36;1.30]
Hemorrhagic stroke	1 (0.1) [0.00;0.17]	1 (0.1) [0.00;0.20]
SEEs	1 (0.1) [0.00;0.17]	1 (0.1) [0.00;0.20]
TIA	4 (0.2) [0.00;0.46]	2 (0.1) [0.00;0.33]
MACE	28 (1.7) [1.04;2.27]	32 (2.2) [1.46;3.02]
VTE episodes	0 (0) [0.00;0.00]	2 (0.1) [0.00;0.33]
ACS	11 (0.6) [0.27;1.03]	5 (0.3) [0.04;0.65]
Hospitalizations related to cardiovascular conditions	119 (8.0) [6.53;9.40]	106 (8.6) [6.93;10.19]

* Percentages are based on the total number of patients with non-missing observations (excluding patients with missing or unknown data). ACS, acute coronary syndrome; CV, cardiovascular; MACE, major adverse cardiovascular events; SEEs, systemic embolic events; TIA, transient ischemic attack; VTE, venous thromboembolism.

**Table 7:** Clinical outcomes by age group at baseline (FAS).

Relevant Clinical Event Annualized Event Rates N (%/Year) [95% CI] *	<65 Years (*n* = 157)	Between 65 and 75 Years (*n* = 304)	≥75 Years (*n* = 470)
All-cause death	9 (1.6) [0.57;2.70]	14 (1.3) [0.60;1.93]	98 (6.5) [5.20;7.77]
CV death	4 (0.7) [0.01;1.43]	4 (0.4) [0.01;0.72]	40 (2.6) [1.83;3.47]
Any bleeding events	10 (1.9) [0.71;3.04]	28 (2.7) [1.69;3.68]	82 (6.0) [4.72;7.32]
Major bleeding events	2 (0.4) [0.00;0.86]	8 (0.7) [0.23;1.24]	37 (2.5) [1.71;3.34]
ICH events	0 (0.0) [0.00;0.00]	2 (0.2) [0.00;0.43]	3 (0.2) [0.00;0.42]
Gastrointestinal bleeding events	4 (0.7) [0.01;1.45]	10 (0.9) [0.35;1.50]	39 (2.7) [1.85;3.54]
Major gastrointestinal bleeding events	1 (0.2) [0.00;0.54]	3 (0.3) [0.00;0.58]	20 (1.3) [0.76;1.94]
Stroke	0 (0.0) [0.00;0.00]	7 (0.6) [0.17;1.11]	15 (1.0) [0.50;1.51]
Ischemic stroke	0 (0.0) [0.00;0.00]	5 (0.5) [0.06;0.86]	14 (0.9) [0.45;1.42]
Hemorrhagic stroke	0 (0.0) [0.00;0.00]	2 (0.2) [0.00;0.43]	0 (0.0) [0.00;0.00]
SEEs	0 (0.0) [0.00;0.00]	0 (0.0) [0.00;0.00]	2 (0.1) [0.00;0.32]
TIA	1 (0.2) [0.00;0.54]	2 (0.2) [0.00;0.43]	3 (0.2) [0.00;0.42]
MACE	3 (0.5) [0.00;1.16]	11 (1.0) [0.41;1.61]	46 (3.1) [2.21;4.00]
VTE episodes	0 (0.0) [0.00;0.00]	0 (0.0) [0.00;0.00]	2 (0.1) [0.00;0.32]
ACS	2 (0.4) [0.00;0.87]	4 (0.4) [0.01;0.72]	10 (0.7) [0.25;1.08]
Hospitalizations related to cardiovascular conditions	33 (6.8) [4.48;9.11]	59 (6.1) [4.54;7.65]	133 (10.4) [8.63;12.17]

* Percentages are based on the total number of patients with non-missing observations (excluding patients with missing or unknown data). ACS, acute coronary syndrome; CV, cardiovascular; MACE, major adverse cardiovascular events; SEEs, systemic embolic events; TIA, transient ischemic attack; VTE, venous thromboembolism.

**Table 8:** Clinical outcomes by renal function at baseline (FAS).

Relevant Clinical Event Annualized Event Rates N (%/Year) [95% CI] *	≤50 mL/min (*n* = 184)	>50 and ≤80 mL/min (*n* = 341)	>80 mL/min (*n* = 309)
All-cause death	48 (8.5) [6.08;10.88]	50 (4.4) [3.18;5.62]	18 (1.6) [0.87;2.37]
CV death	19 (3.4) [1.85;4.87]	19 (1.7) [0.92;2.42]	8 (0.7) [0.22;1.22]
Any bleeding events	39 (7.7) [5.26;10.07]	46 (4.4) [3.15;5.71]	32 (3.1) [2.01;4.13]
Major bleeding events	20 (3.7) [2.06;5.28]	21 (1.9) [1.09;2.73]	6 (0.5) [0.11;0.98]
ICH events	4 (0.7) [0.01;1.41]	0 (0.0) [0.00;0.00]	1 (0.1) [0.00;0.27]
Gastrointestinal bleeding events	15 (2.8) [1.36;4.15]	24 (2.2) [1.32;3.08]	12 (1.1) [0.48;1.72]
Major gastrointestinal bleeding events	7 (1.3) [0.33;2.18]	14 (1.3) [0.60;1.92]	3 (0.3) [0.00;0.58]
Stroke	10 (1.8) [0.68;2.88]	5 (0.4) [0.05;0.83]	6 (0.5) [0.11;0.98]
Ischemic stroke	9 (1.6) [0.55;2.64]	5 (0.4) [0.05;0.83]	4 (0.4) [0.01;0.72]
Hemorrhagic stroke	1 (0.2) [0.00;0.52]	0 (0.0) [0.00;0.00]	1 (0.1) [0.00;0.27]
SEEs	1 (0.2) [0.00;0.52]	1 (0.1) [0.00;0.26]	0 (0.0) [0.00;0.00]
TIA	4 (0.7) [0.01;1.41]	1 0.1 [0.00;0.26]	1 (0.1) [0.00;0.27]
MACE	23 (4.2) [2.46;5.85]	22 (2.0) [1.14;2.79]	13 (1.2) [0.54;1.84]
VTE episodes	0 (0.0) [0.00;0.00]	2 (0.2) [0.00;0.42]	0 (0.0) [0.00;0.00]
ACS	5 (0.9) [0.11;1.69]	6 (0.5) [0.11;0.96]	5 (0.5) [0.06;0.85]
Hospitalizations related to cardiovascular conditions	57 (12.1) [9.00;15.30]	90 (9.4) [7.47;11.36]	69 (7.1) [5.45;8.82]

* Percentages are based on the total number of patients with non-missing observations (excluding patients with missing or unknown data). ACS, acute coronary syndrome; CV, cardiovascular; MACE, major adverse cardiovascular events; SEEs, systemic embolic events; TIA, transient ischemic attack; VTE, venous thromboembolism.

**Table 9:** Clinical outcomes by CHA2DS2-VASc risk group (derived) at baseline (FAS).

Relevant Clinical Event Annualized Event Rates N (%/Year) [95% CI] *	Low (0–1 pt) (*n* = 83)	Moderate (2–4 pts) (*n* = 657)	High (>4 pts) (*n* = 176)
All-cause death	3 (1.1) [0.00;2.27]	79 (3.5) [2.72;4.25]	36 (6.3) [4.26;8.39]
CV death	2 (0.7) [0.00;1.70]	29 (1.3) [0.81;1.74]	16 (2.8) [1.43;4.19]
Any bleeding events	3 (1.1) [0.00;2.34]	87 (4.1) [3.27;5.00]	30 (5.9) [3.78;7.99]
Major bleeding events	0 (0.0) [0.00;0.00]	34 (1.5) [1.02;2.04]	13 (2.4) [1.08;3.64]
ICH events	0 (0.0) [0.00;0.00]	4 (0.2) [0.00;0.35]	1 (0.2) [0.00;0.52]
Gastrointestinal bleeding events	0 (0.0) [0.00;0.00]	36 (1.6) [1.10;2.17]	17 (3.1) [1.64;4.62]
Major gastrointestinal bleeding events	0 (0.0) [0.00;0.00]	13 (0.6) [0.26;0.89]	11 (2.0) [0.81;3.17]
Stroke	1 (0.4) [0.00;1.06]	14 (0.6) [0.30;0.95]	7 (1.3) [0.33;2.18]
Ischemic stroke	1 (0.4) [0.00;1.06]	12 (0.5) [0.23;0.83]	6 (1.1) [0.21;1.93]
Hemorrhagic stroke	0 (0.0) [0.00;0.00]	2 (0.1) [0.00;0.21]	0 (0.0) [0.00;0.00]
SEEs	0 (0.0) [0.00;0.00]	1 (<0.1) [0.00;0.13]	1 (0.2) [0.00;0.52]
TIA	0 (0.0) [0.00;0.00]	5 (0.2) [0.03;0.41]	1 (0.2) [0.00;0.52]
MACE	2 (0.7) [0.00;1.71]	33 (1.5) [0.97;1.97]	25 (4.6) [2.78;6.37]
VTE episodes	0 (0.0) [0.00;0.00]	2 (0.1) [0.00;0.21]	0 (0.0) [0.00;0.00]
ACS	0 (0.0) [0.00;0.00]	11 (0.5) [0.20;0.78]	5 (0.9) [0.11;1.68]
Hospitalizations related to cardiovascular conditions	9 (3.4) [1.19;5.66]	160 (8.1) [6.87;9.39]	53 (11.5) [8.42;14.63]

* Percentages are based on the total number of patients with non-missing observations (excluding patients with missing or unknown data). ACS, acute coronary syndrome; CV, cardiovascular; MACE, major adverse cardiovascular events; SEEs, systemic embolic events; TIA, transient ischemic attack; VTE, venous thromboembolism.

## Data Availability

The datasets presented in this article are not readily available due to technical/time limitations. Requests to access the datasets should be directed to https://vivli.org/ourmember/daiichi-sankyo/.

## Supplementary Materials

The following supporting information can be downloaded at https://www.mdpi.com/article/10.3390/jcm15114085/s1. Table S1: Distribution of patients according to the edoxaban SmPC by initial edoxaban dose at study start (baseline analysis set); Table S2: Baseline characteristics of patients enrolled in Spain and Portugal. Results split by edoxaban 60 mg and 30 mg doses in line with and not in line with SmPC recommendations at baseline (full analysis set); Table S3: Baseline characteristics of patients enrolled in Spain and Portugal. Results split by sex (full analysis set); Table S4: Baseline characteristics of patients enrolled in Spain and Portugal. Results split by age at baseline (full analysis set); Table S5: Baseline characteristics of patients enrolled in Spain and Portugal. Results split by renal function at baseline (full analysis set); Table S6: Baseline characteristics of patients enrolled in Spain and Portugal. Results split by CHA2DS2-VASc Risk Group (derived) at baseline (full analysis set); Figure S1: Kaplan–Meier plot for time-to-death for any cause by sex for patients enrolled in Spain and Portugal (full analysis set); Figure S2: Kaplan–Meier plot for time-to-cardiovascular death by sex for patients enrolled in Spain and Portugal (full analysis set); Figure S3: Kaplan–Meier plot for time-to-first occurrence of stroke by sex for patients enrolled in Spain and Portugal (full analysis set); Figure S4: Kaplan–Meier plot for time-to-first major bleeding event by sex for patients enrolled in Spain and Portugal (full analysis set); Figure S5: Kaplan–Meier plot for time-to-first occurrence of ICH by sex for patients enrolled in Spain and Portugal (full analysis set); Figure S6: Kaplan–Meier plot for time-to-death for any cause by age at baseline for patients enrolled in Spain and Portugal (full analysis set); Figure S7: Kaplan–Meier plot for time-to-cardiovascular death by age at baseline for patients enrolled in Spain and Portugal (full analysis set); Figure S8: Kaplan–Meier plot for time-to-first occurrence of stroke by age at baseline for patients enrolled in Spain and Portugal (full analysis set); Figure S9: Kaplan–Meier plot for time-to-first major bleeding event by age at baseline for patients enrolled in Spain and Portugal (full analysis set); Figure S10: Kaplan–Meier plot for time-to-first occurrence of ICH by age at baseline for patients enrolled in Spain and Portugal (full analysis set); Figure S11: Kaplan–Meier plot for time-to-death for any cause by renal function at baseline for patients enrolled in Spain and Portugal (full analysis set); Figure S12: Kaplan–Meier plot for time-to-cardiovascular death by renal function at baseline for patients enrolled in Spain and Portugal (full analysis set); Figure S13: Kaplan–Meier plot for time-to-first occurrence of stroke by renal function at baseline for patients enrolled in Spain and Portugal (full analysis set): Figure S14: Kaplan–Meier plot for time-to-first major bleeding event by renal function at baseline for patients enrolled in Spain and Portugal (full analysis set): Figure S15: Kaplan–Meier plot for time-to-first occurrence of ICH by renal function at baseline for patients enrolled in Spain and Portugal (full analysis set); Figure S16: Kaplan–Meier plot for time-to-death for any cause by CHA2DS2-VASc Risk Group (derived) at baseline for patients enrolled in Spain and Portugal (full analysis set); Figure S17: Kaplan–Meier plot for time-to-cardiovascular death by CHA2DS2-VASc Risk Group (derived) at baseline for patients enrolled in Spain and Portugal (full analysis set): Figure S18: Kaplan–Meier plot for time-to-first occurrence of stroke by CHA2DS2-VASc Risk Group (derived) at baseline for patients enrolled in Spain and Portugal (full analysis set); Figure S19: Kaplan–Meier plot for time-to-first major bleeding event by CHA2DS2-VASc Risk Group (derived) at baseline for patients enrolled in Spain and Portugal (full analysis set); Figure S20: Kaplan–Meier plot for time-to-first occurrence of ICH by CHA2DS2-VASc Risk Group (derived) at baseline for patients enrolled in Spain and Portugal (full analysis set).
