# Supplementary material for: Edoxaban for Stroke Prevention in Atrial Fibrillation in Spain and Portugal: 4-Year Follow-Up of the Observational ETNA-AF-Europe Study
Source: J Clin Med. 2026 May 25;15(11):4085. doi: 10.3390/jcm15114085 (PMC13257549; doi:10.3390/jcm15114085)
Supplement: Supplementary file 1 [file jcm-15-04085-s001.zip › jcm-4221442-supplementary.pdf]

SUPPLEMENTARY DATA

TABLES

Table S1. Distribution of patients according to the edoxaban SmPC by initial edoxaban dose at study start (baseline analysis set).

**Dosing, *n* (%)**	**Overall (*n* = 950)**	**Edoxaban 60 mg (*n* = 714)**	**Edoxaban 30 mg (*n* = 221)**
60 mg - once daily and without any fulfilled reason for dose adjustment (i.e., no moderate-severe renal impairment, and no body weight ≤ 60 kg, and no concomitant use of P-gp inhibitors) and also no missing information	538 (56.6)	538 (75.4)	0
60 mg - with documented reason for dose reduction	81 (8.5)	81 (11.3)	0
with moderate-severe renal impairment	56 (5.9)	56 (7.8)	0
with body weight <= 60kg	31 (3.3)	31 (4.3)	0
with concomitant use of P-gp inhibitors	5 (0.5)	5 (0.7)	0
30 mg - once daily and at least 1 documented reason for dose adjustment (i.e., moderate-severe renal impairment, or body weight ≤ 60kg, or concomitant use of P-gp inhibitors)	162 (17.1)	0	162 (73.3)
30 mg - without any fulfilled reason for dose reduction (also no missing)	45 (4.7)	0	45 (20.4)
Patient with insufficient data to judge dose adequacy			
60 mg - with missing information for at least one potential reasons for dose reduction and no other reason fulfilled	95 (10.0)	95 (13.3)	0
30 mg - with missing information for at least one potential reason for dose reduction and no other reason for dose adjustment fulfilled	14 (1.5)	0	14 (6.3)

Percentages are based on the total number of patients indicated in the column's headers. P-gp, P-glycoprotein.

Table S2. Baseline characteristics of patients enrolled in Spain and Portugal. Results split by edoxaban 60 mg and 30 mg doses in line and not in line with SmPC recommendations at baseline (full analysis set).

Characteristic, *n* (%) or median (IQR)	Patients with SmPC recommendation for 60 mg		Patients with SmPC recommendation for 30 mg		Patients with no confirmation on reason for choice of dosing	
	60 mg recommended dose (*n* = 530)	30 mg non-recommended dose (*n* = 45)	30 mg recommended dose (*n* = 159)	60 mg non-recommended dose (*n* = 79)	60 mg (*n* = 92)	30 mg (*n* = 13)
Age, years	72.0 (66.0, 79.0)	76.0 (67.0, 80.0)	82.0 (78.0, 87.0)	81.0 (75.0, 86.0)	71.5 (66.0, 77.5)	82.0 (79.0, 86.0)
Male	321 (60.6)	24 (53.3)	59 (37.1)	27 (34.2)	62 (67.4)	9 (69.2)
Weight, kg	80.0 (72.0, 89.0)	85.0 (72.0, 92.0)	62.0 (55.0, 74.0)	64.5 (59.5, 71.0)	80.0 (75.0, 92.0)	83.0 (82.0, 89.0)
Body mass index, kg/m2	29.0 (26.6, 31.6)	30.9 (27.2, 33.7)	25.3 (22.8, 28.6)	24.9 (22.4, 27.8)	27.3 (25.9, 29.9)	32.8 (31.5, 33.8)
Overweight (≥25 and <30 kg/m2)	249 (47.5)	14 (31.1)	53 (34.6)	25 (33.8)	17 (65.4)	0
Obese (≥30 kg/m2)	211 (40.3)	25 (55.6)	30 (19.6)	12 (16.2)	6 (23.1)	4 (100)
CrCl [ml/min]						
<15	0	0	0	0	0	0
≥15 – ≤30	0	0	20 (12.9)	2 (2.6)	0	0
>30 – ≤50	0	0	106 (68.4)	53 (68.8)	0	0
>50 – ≤80	247 (47.7)	37 (82.2)	24 (15.5)	18 (23.4)	7 (58.3)	2 (66.7)
>80	271 (52.3)	8 (17.8)	5 (3.2)	4 (5.2)	5 (41.7)	1 (33.3)
History of renal impairment	247 (46.6)	37 (82.2)	150 (96.8)	73 (93.6)	7 (58.3)	2 (66.7)
SBP, mmHg	130.0 (120.0, 142.0)	134.5 (125.0, 150.0)	131.5 (120.0, 145.0)	129.0 (115.0, 140.0)	125.5 (120.0, 140.0)	129.0 (120.0, 140.0)
Uncontrolled hypertension episode (SBP >160 mmHg)	63 (13.6)	8 (19.0)	18 (13.6)	4 (6.6)	3 (4.3)	1 (11.1)
CHA2DS2-VASc Risk score						
Low (0-1 pt)	61 (11.7)	2 (4.4)	1 (0.6)	1 (1.3)	16 (17.8)	1 (7.7)
Moderate (2-4 pts)	373 (71.7)	35 (77.8)	108 (69.2)	60 (75.9)	62 (68.9)	9 (69.2)
High (>4 pts)	86 (16.5)	8 (17.8)	47 (30.1)	18 (22.8)	12 (13.3)	3 (23.1)
Current smoker	31 (6.2)	2 (5.0)	1 (0.7)	4 (5.6)	3 (6.1)	1 (16.7)
Frailty (investigator-assessed)	34 (6.6)	6 (13.6)	46 (30.7)	16 (21.3)	0	3 (25.0)
Chronic hepatic disease	8 (1.5)	0	3 (1.9)	3 (3.9)	1 (1.2)	0
Type of NVAF						
Paroxysmal	257 (48.5)	19 (42.2)	67 (42.1)	37 (46.8)	58 (63.0)	5 (38.5)
Persistent	122 (23.0)	8 (17.8)	29 (18.2)	20 (25.3)	11 (12.0)	1 (7.7)
Long-standing persistent or permanent	151 (28.5)	18 (40.0)	63 (39.6)	22 (27.8)	23 (25.0)	7 (53.8)
History of stroke	58 (10.9)	1 (2.2)	16 (10.1)	5 (6.3)	7 (7.6)	0

Haemorrhagic Stroke	2 (0.4)	0	2 (1.3)	0	0	0
Ischemic Stroke	54 (10.2)	1 (2.2)	13 (8.2)	5 (6.3)	6 (6.5)	0
Unknown Stroke Type	2 (0.4)	0	1 (0.6)	0	1 (1.1)	0
History of TIA (transient ischaemic stroke)	18 (3.4)	1 (2.2)	9 (5.7)	1 (1.3)	5 (5.4)	0
History of CV disease						
Hypertension	401 (75.7)	39 (86.7)	121 (76.1)	59 (74.7)	68 (73.9)	11 (84.6)
Coronary heart disease	55 (10.4)	13 (28.9)	18 (11.3)	11 (13.9)	2 (2.2)	3 (23.1)
Peripheral artery disease	9 (1.7)	4 (8.9)	2 (1.3)	5 (6.3)	1 (1.1)	0
History of DM	151 (28.5)	12 (26.7)	40 (25.2)	18 (22.8)	18 (19.6)	5 (38.5)
History of COPD	53 (10.0)	1 (2.2)	11 (6.9)	4 (5.1)	1 (1.1)	0

Percentages are based on the total number of patients with non-missing observations (excluding patients with missing and unknown data). The information regarding median (IQR) creatinine clearance, median (IQR) CHA2DS2-VASc Risk score, Modified HAS-BLED score and history of valvular heart diseases is missing for edoxaban 60 mg and 30 mg doses groups in line and not in line with SmPC recommendations COPD, chronic obstructive pulmonary disease; CrCl, creatinine clearance; CV, cardiovascular; DM, diabetes mellitus; IQR, interquartile range; NVAF, non-valvular atrial fibrillation; SBP, systolic blood pressure; TIA, transient ischaemic stroke.

Table S3. Baseline characteristics of patients enrolled in Spain and Portugal. Results split by sex (full analysis set).

Characteristic, *n* (%) or median (IQR)	Male (*n* = 511)	Female (*n* = 420)
Age, years	73.0 (66.0, 81.0)	76.0 (69.5, 82.0)
Weight, kg	80.0 (73.0, 90.0)	70.0 (62.0, 80.0)
Body mass index, kg/m2	27.8 (25.6, 30.8)	28.3 (25.1, 32.0)
Overweight (≥25 and <30 kg/m2)	221 (49.2)	144 (37.1)
Obese (≥30 kg/m2)	142 (31.6)	148 (38.1)
CrCl [ml/min]	75.16 (56.11, 94.70)	64.23 (47.52, 82.42)
<15	0	0
≥15 – ≤30	10 (2.2)	13 (3.4)
>30 – ≤50	65 (14.5)	96 (24.9)
>50 – ≤80	178 (39.7)	163 (42.2)
>80	195 (43.5)	114 (29.5)
History of renal impairment	254 (50.5)	272 (65.7)
SBP, mmHg	130.0 (120.0, 140.0)	131.0 (120.0, 143.0)
Uncontrolled hypertension episode (SBP >160 mmHg)	53 (12.4)	48 (13.2)
CHA2DS2-VASc Risk score	3.0 (2.0, 4.0)	4.0 (3.0, 5.0)
Low (0-1 pt)	76 (15.2)	7 (1.7)
Moderate (2-4 pts)	362 (72.5)	295 (70.7)
High (>4 pts)	61 (12.2)	115 (27.6)
Modified HAS-BLED score	2.0 (1.0, 2.0)	2.0 (1.0, 2.0)
Low (0-1 pt)	161 (39.2)	109 (30.4)
Medium (2 pts)	167 (40.6)	188 (52.4)
High (>2 pts)	83 (20.2)	62 (17.3)
Current smoker	31 (6.9)	11 (2.9)
Frailty (investigator-assessed)	40 (8.1)	67 (16.7)
Chronic hepatic disease	10 (2.0)	6 (1.5)
Type of NVAF		
Paroxysmal	236 (46.2)	211 (50.2)
Persistent	117 (22.9)	79 (18.8)
Long-standing persistent or permanent	158 (30.9)	130 (31.0)
History of stroke	51 (10.0)	36 (8.6)
Haemorrhagic Stroke	2 (0.4)	2 (0.5)
Ischemic Stroke	47 (9.2)	32 (7.6)
Unknown Stroke Type	2 (0.4)	2 (0.5)
History of TIA (transient ischaemic stroke)	21 (4.1)	14 (3.3)
History of CV disease		
Hypertension	390 (76.3)	320 (76.2)
Coronary heart disease	86 (17.0)	17 (4.1)
Peripheral artery disease	21 (4.2)	0
Valvular heart disease	50 (9.9)	55 (13.1)
History of DM	162 (32.1)	85 (20.3)
History of COPD	60 (11.8)	13 (3.1)

Percentages are based on the total number of patients with non-missing observations (excluding patients with missing and unknown data). COPD, chronic obstructive pulmonary disease; CrCl, creatinine clearance; CV, cardiovascular; DM, diabetes mellitus; HAS-BLED, hypertension, abnormal liver/renal function, stroke history, bleeding history or predisposition, labile INR, elderly, drug/alcohol usage; IQR, interquartile range; NVAF, non-valvular atrial fibrillation; SBP, systolic blood pressure; TIA, transient ischaemic stroke.

Table S4. Baseline characteristics of patients enrolled in Spain and Portugal. Results split by age at baseline (full analysis set).

Characteristic, *n* (%) or median (IQR)	<65 years (*n* = 157)	>=65 and <75 years (*n* = 304)	>=75 years (*n* = 470)
Male	110 (70.1)	168 (55.3)	233 (49.6)
Weight, kg	84.0 (76.0, 95.0)	78 (70.0, 87.0)	72.0 (63.0, 82.0)
Body mass index, kg/m2	29.1 (26.45, 31.85)	28.2 (25.50, 31.60)	27.5 (24.70, 30.80)
Overweight (≥25 and <30 kg/m2)	67 (46.5)	117 (42.7)	181 (43.2)
Obese (≥30 kg/m2)	62 (43.1)	99 (36.1)	129 (30.8)
CrCl [ml/min]	101.28 (84.01, 117.84)	81.17 (68.42, 95.88)	56.42 (43.17, 69.79)
<15	0	0	0
≥15 – ≤30	1 (0.7)	2 (0.7)	20 (4.7)
>30 – ≤50	4 (2.9)	16 (6.0)	141 (33.0)
>50 – ≤80	22 (15.7)	110 (41.2)	209 (48.9)
>80	113 (80.7)	139 (52.1)	57 (13.3)
History of renal impairment	27 (17.5)	128 (42.5)	371 (80.3)
SBP, mmHg	130 (120.0, 141.5)	130 (120.0, 140.0)	132 (120.0, 143.0)
Uncontrolled hypertension episode (SBP >160 mmHg)	14 (10.6)	31 (11.6)	56 (14.3)
CHA2DS2-VASc Risk score	2.0 (1.0, 2.0)	3.0 (2.0, 3.0)	4.0 (3.0, 5.0)
Low (0-1 pt)	55 (36.4)	28 (9.4)	0
Moderate (2-4 pts)	92 (60.9)	246 (82.6)	319 (68.3)
High (>4 pts)	4 (2.6)	24 (8.1)	148 (31.7)
Modified HAS-BLED score	1.0 (0.0, 1.0)	2.0 (1.0, 2.0)	2.0 (2.0, 3.0)
Low (0-1 pt)	108 (84.4)	116 (44.6)	46 (12.0)
Medium (2 pts)	14 (10.9)	107 (41.2)	234 (61.3)
High (>2 pts)	6 (4.7)	37 (14.2)	102 (26.7)
Current smoker	17 (12.2)	20 (7.4)	5 (1.2)
Frailty (investigator-assessed)	5 (3.3)	9 (3.0)	93 (20.7)
Chronic hepatic disease	5 (3.3)	6 (2.0)	5 (1.1)
Type of NVAF			
Paroxysmal	92 (58.6)	159 (52.3)	196 (41.7)
Persistent	37 (23.6)	68 (22.4)	91 (19.4)
Long-standing persistent or permanent	28 (17.8)	77 (25.4)	183 (39.0)
History of stroke	17 (10.8)	21 (6.9)	49 (10.4)
Haemorrhagic Stroke	0	0	4 (0.9)
Ischemic Stroke	17 (10.8)	19 (6.3)	43 (9.1)
Unknown Stroke Type	0	2 (0.7)	2 (0.4)
History of TIA (transient ischaemic stroke)	8 (5.1)	7 (2.3)	20 (4.3)
History of CV disease			
Hypertension	115 (73.2)	220 (72.4)	375 (79.8)
Coronary heart disease	17 (10.9)	27 (9.0)	59 (12.6)
Peripheral artery disease	2 (1.3)	5 (1.7)	14 (3.0)
Valvular heart disease	5 (3.2)	27 (9.0)	73 (15.6)
History of DM	47 (30.7)	73 (24.3)	127 (27.0)
History of COPD	10 (6.5)	24 (8.0)	39 (8.3)

Percentages are based on the total number of patients with non-missing observations (excluding patients with missing and unknown data). COPD, chronic obstructive pulmonary disease; CrCl, creatinine clearance; CV, cardiovascular; DM, diabetes mellitus; HAS-BLED, hypertension, abnormal liver/renal function, stroke history, bleeding history or predisposition, labile INR, elderly, drug/alcohol usage; IQR, interquartile range; NVAF, non-valvular atrial fibrillation; SBP, systolic blood pressure; TIA, transient ischaemic stroke.

Table S5. Baseline characteristics of patients enrolled in Spain and Portugal. Results split by renal function at baseline (full analysis set).

**Characteristic, *n* (%) or median (IQR)**	**<=50mL/min (*n* = 184)**	**>50 and <=80mL/min (*n* = 341)**	**>80mL/min (*n* = 309)**
Age, years	84.0 (79.0, 88.0)	77.0 (72.0, 81.0)	68 (61.0, 72.0)
Male	75 (40.8)	178 (52.2)	195 (63.1)
Weight, kg	66.0 (59.0, 74.0)	74.0 (67.0, 83.0)	83.0 (77.0, 93.0)
Body mass index, kg/m2	25.9 (23.4, 28.8)	27.4 (25.0, 30.5)	29.8 (27.6, 33.1)
Overweight (≥25 and <30 kg/m2)	68 (38.6)	151 (45.8)	133 (43.6)
Obese (≥30 kg/m2)	37 (21.0)	97 (29.4)	149 (48.9)
CrCl [ml/min]	40.78 (34.99, 45.38)	64.81 (57.01, 72.61)	95.96 (87.42, 110.16)
<15	0	0	0
≥15 – ≤30	23 (12.5)	0	0
>30 – ≤50	161 (87.5)	0	0
>50 – ≤80	0	341 (100)	0
>80	0	0	309 (100)
History of renal impairment	184 (100)	341 (100)	0
SBP, mmHg	130.0 (120.0, 143.0)	131.0 (120.0, 142.0)	130.0 (120.0, 142.0)
Uncontrolled hypertension episode (SBP >160 mmHg)	18 (11.9)	47 (15.6)	34 (12.9)
CHA2DS2-VASc Risk score	4.0 (3.0, 5.0)	4.0 (3.0, 4.0)	2.0 (2.0, 3.0)
Low (0-1 pt)	0	17 (5.0)	52 (17.2)
Moderate (2-4 pts)	128 (70.7)	238 (70.6)	221 (73.2)
High (>4 pts)	53 (29.3)	82 (24.3)	29 (9.6)
Modified HAS-BLED score	2.0 (2.0, 3.0)	2.0 (2.0, 3.0)	1.0 (1.0, 1.0)
Low (0-1 pt)	0	10 (3.3)	205 (79.2)
Medium (2 pts)	108 (73.0)	194 (64.9)	44 (17.0)
High (>2 pts)	40 (27.0)	95 (31.8)	10 (3.9)
Current smoker	1 (0.6)	13 (4.1)	24 (8.2)
Frailty (investigator-assessed)	54 (30.9)	38 (11.6)	14 (4.7)
Chronic hepatic disease	3 (1.7)	6 (1.8)	6 (2.0)
Type of NVAF			
Paroxysmal	74 (40.2)	153 (44.9)	158 (51.1)
Persistent	38 (20.7)	76 (22.3)	71 (23.0)
Long-standing persistent or permanent	72 (39.2)	112 (32.9)	80 (25.9)
History of stroke	15 (8.2)	40 (11.7)	27 (8.7)
Haemorrhagic Stroke	2 (1.1)	2 (0.6)	0
Ischemic Stroke	12 (6.5)	36 (10.6)	27 (8.7)
Unknown Stroke Type	1 (0.5)	2 (0.6)	0
History of TIA (transient ischaemic stroke)	9 (4.9)	11 (3.2)	10 (3.2)
History of CV disease			
Hypertension	149 (81.0)	263 (77.1)	225 (72.8)
Coronary heart disease	27 (14.8)	43 (12.7)	32 (10.4)
Peripheral artery disease	6 (3.3)	10 (3.0)	4 (1.3)
Valvular heart disease	32 (17.5)	41 (12.1)	28 (9.1)
History of DM	52 (28.3)	90 (26.5)	85 (28.0)
History of COPD	9 (4.9)	39 (11.5)	24 (7.9)

Percentages are based on the total number of patients with non-missing observations (excluding patients with missing and unknown data). COPD, chronic obstructive pulmonary disease; CrCl, creatinine clearance; CV, cardiovascular; DM, diabetes mellitus; HAS-BLED, hypertension, abnormal liver/renal function, stroke history, bleeding history or predisposition, labile INR, elderly, drug/alcohol usage; IQR, interquartile range; NVAF, non-valvular atrial fibrillation; SBP, systolic blood pressure; TIA, transient ischaemic stroke.

Table S6. Baseline characteristics of patients enrolled in Spain and Portugal. Results split by CHA2DS2-VASc Risk Group (derived) at baseline (full analysis set)

**Characteristic, *n* (%) or median (IQR)**	**Low (0-1 pt) (*n* = 83)**	**Moderate (2-4 pts) (*n* = 657)**	**High (>4 pts) (*n* = 176)**
Age, years	63.0 (57.0, 68.0)	74.0 (68.0, 81.0)	80.0 (76.5, 84.0)
Male	76 (91.6)	362 (55.1)	61 (34.7)
Weight, kg	83.0 (76.0, 90.0)	76.0 (67.0, 86.0)	74.0 (63.5, 82.5)
Body mass index, kg/m2	27.9 (25.7, 31.6)	28.1 (25.3, 31.2)	28.4 (25.8, 31.9)
Overweight (≥25 and <30 kg/m2)	33 (45.8)	257 (43.3)	67 (42.7)
Obese (≥30 kg/m2)	26 (36.1)	200 (33.7)	60 (38.2)
CrCl [ml/min]	91.72 (79.85, 109.87)	70.62 (53.33, 89.49)	58.33 (45.65, 73.81)
<15	0	0	0
≥15 – ≤30	0	19 (3.2)	4 (2.4)
>30 – ≤50	0	109 (18.6)	49 (29.9)
>50 – ≤80	17 (24.6)	238 (40.5)	82 (50.0)
>80	52 (75.4)	221 (37.6)	29 (17.7)
History of renal impairment	17 (21.3)	366 (56.7)	136 (77.3)
SBP, mmHg	127.5 (119.0, 137.0)	130.0 (120.0, 142.0)	132.0 (120.0, 143.0)
Uncontrolled hypertension episode (SBP >160 mmHg)	2 (2.9)	71 (12.6)	27 (17.8)
CHA2DS2-VASc Risk score	1.0 (1.0, 1.0)	3.0 (2.0, 4.0)	5.0 (5.0, 6.0)
Modified HAS-BLED score	1.0 (0.0, 1.0)	2.0 (1.0, 2.0)	2.0 (2.0, 3.0)
Low (0-1 pt)	54 (81.8)	198 (36.2)	17 (11.3)
Medium (2 pts)	11 (16.7)	275 (50.3)	65 (43.0)
High (>2 pts)	1 (1.5)	74 (13.5)	69 (45.7)
Current smoker	9 (12.7)	30 (5.1)	1 (0.6)
Frailty (investigator-assessed)	1 (1.3)	67 (10.6)	38 (22.6)
Chronic hepatic disease	1 (1.3)	12 (1.9)	3 (1.7)
Type of NVAF			
Paroxysmal	48 (57.8)	323 (49.2)	70 (39.8)
Persistent	18 (21.7)	142 (21.6)	31 (17.6)
Long-standing persistent or permanent	17 (20.5)	192 (29.2)	75 (42.6)
History of stroke	0	24 (3.7)	61 (34.7)
Haemorrhagic Stroke	0	0	4 (2.3)
Ischemic Stroke	0	23 (3.5)	54 (30.7)
Unknown Stroke Type	0	1 (0.2)	3 (1.7)
History of TIA (transient ischaemic stroke)	0	9 (1.4)	26 (14.8)
History of CV disease			
Hypertension	31 (37.3)	501 (76.3)	165 (93.8)
Coronary heart disease	3 (3.6)	74 (11.3)	22 (12.5)
Peripheral artery disease	0	12 (1.8)	9 (5.1)
Valvular heart disease	3 (3.6)	63 (9.6)	35 (19.9)
History of DM	3 (3.6)	138 (21.0)	103 (58.5)
History of COPD	5 (6.1)	55 (8.4)	11 (6.3)

Percentages are based on the total number of patients with non-missing observations (excluding patients with missing and unknown data). COPD, chronic obstructive pulmonary disease; CrCl, creatinine clearance; CV, cardiovascular; DM, diabetes mellitus; HAS-BLED, hypertension, abnormal liver/renal function, stroke history, bleeding history or predisposition, labile INR, elderly, drug/alcohol usage; IQR, interquartile range; NVAF, non-valvular atrial fibrillation; SBP, systolic blood pressure; TIA, transient ischaemic stroke.

FIGURES

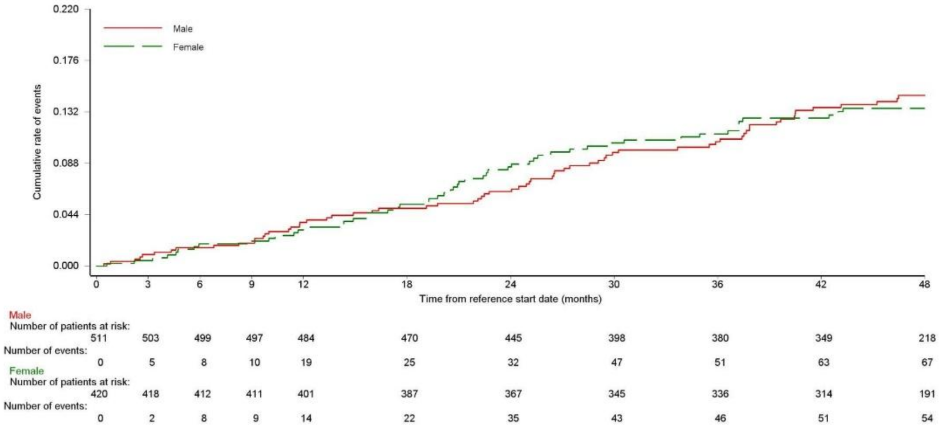

Figure S1. Kaplan-Meier plot for time-to-death for any cause by sex for patients enrolled in Spain and Portugal (full analysis set)

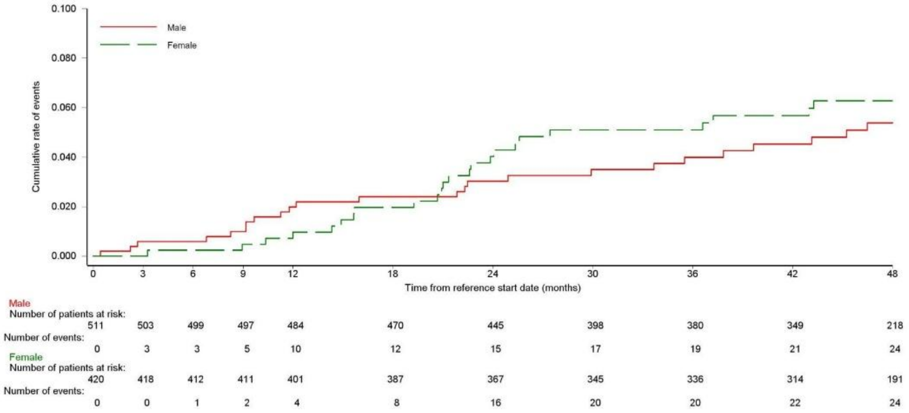

Figure S2. Kaplan-Meier plot for time-to-cardiovascular death by sex for patients enrolled in Spain and Portugal (full analysis set)

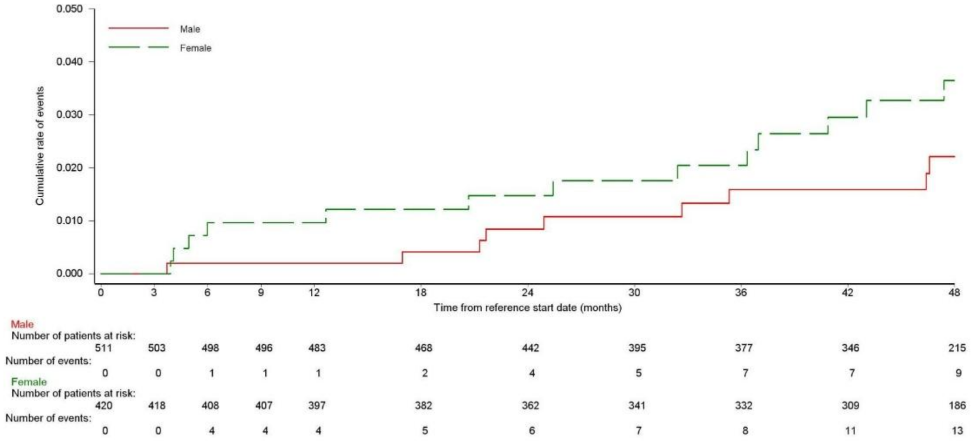

Figure S3. Kaplan-Meier plot for time-to-first occurrence of stroke by sex for patients enrolled in Spain and Portugal (full analysis set)

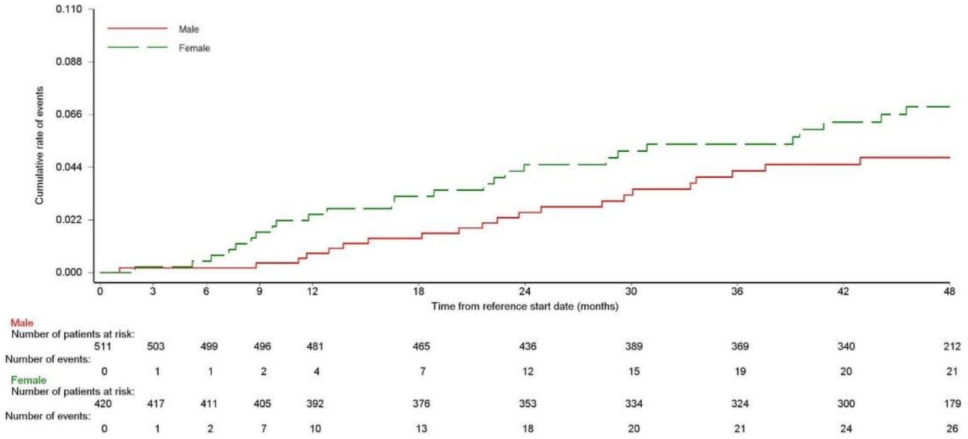

Figure S4. Kaplan-Meier plot for time-to-first major bleeding event by sex for patients enrolled in Spain and Portugal (full analysis set)

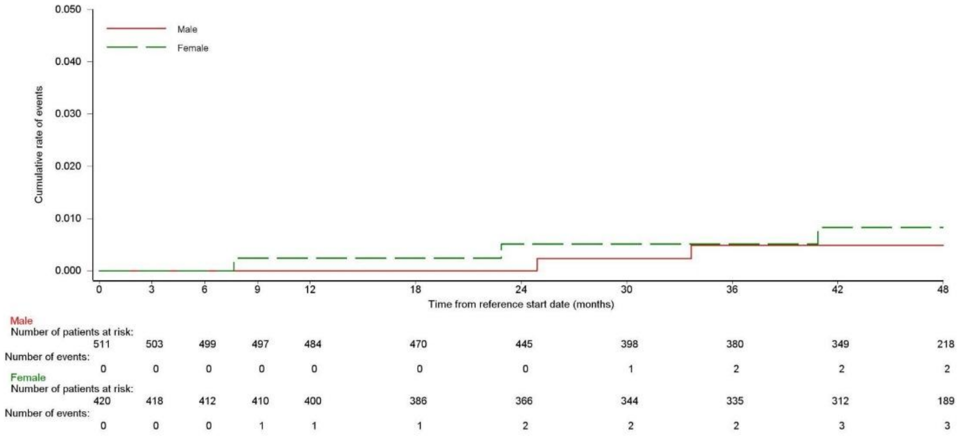

Figure S5. Kaplan-Meier plot for time-to-first occurrence of ICH by sex for patients enrolled in Spain and Portugal (full analysis set)

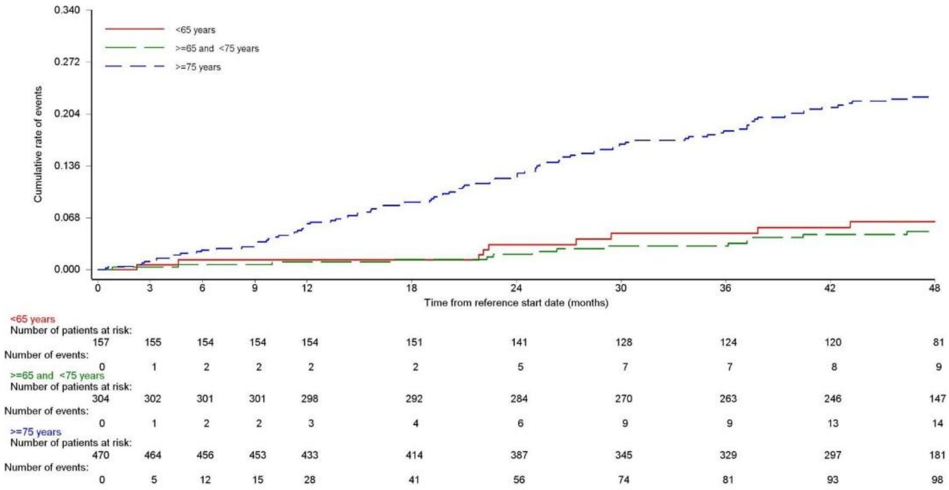

Figure S6. Kaplan-Meier plot for time-to-death for any cause by age at baseline for patients enrolled in Spain and Portugal (full analysis set)

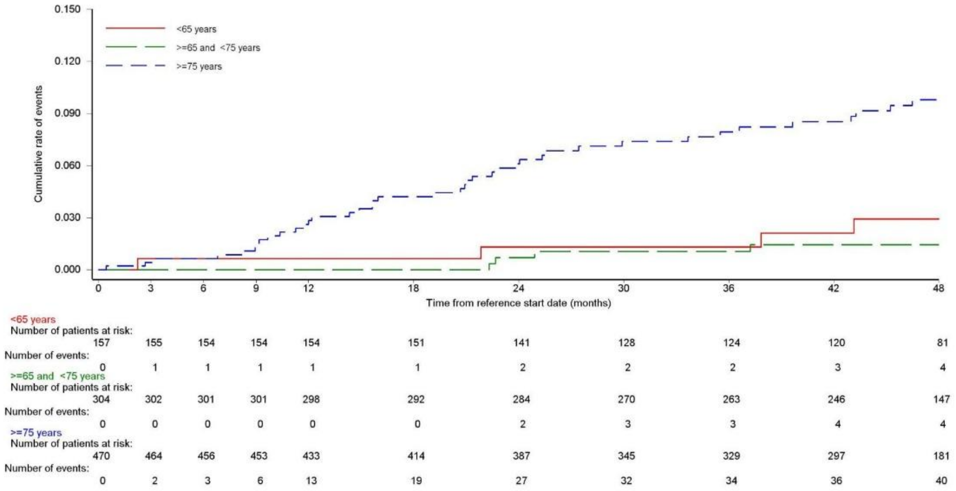

Figure S7. Kaplan-Meier plot for time-to-cardiovascular death by age at baseline for patients enrolled in Spain and Portugal (full analysis set)

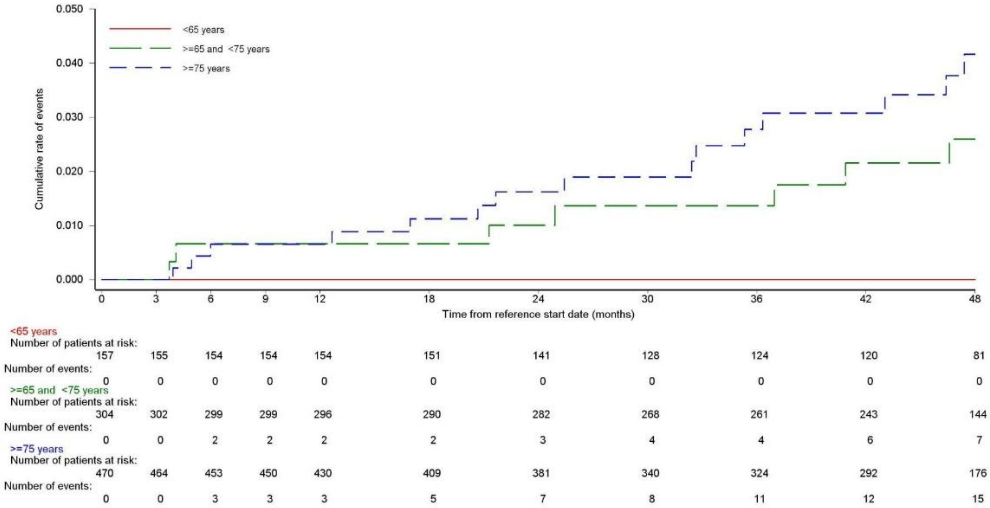

Figure S8. Kaplan-Meier plot for time-to-first occurrence of stroke by age at baseline for patients enrolled in Spain and Portugal (full analysis set)

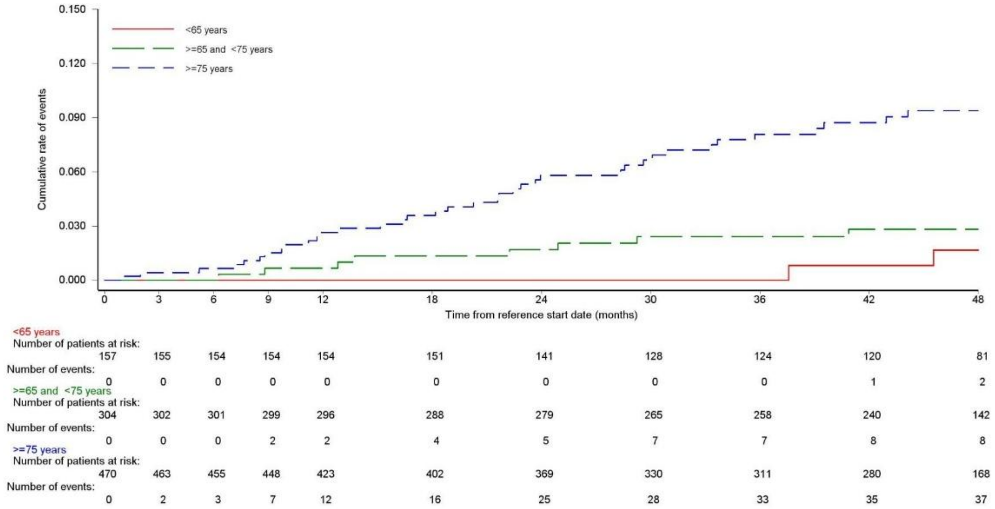

Figure S9. Kaplan-Meier plot for time-to-first major bleeding event by age at baseline for patients enrolled in Spain and Portugal (full analysis set)

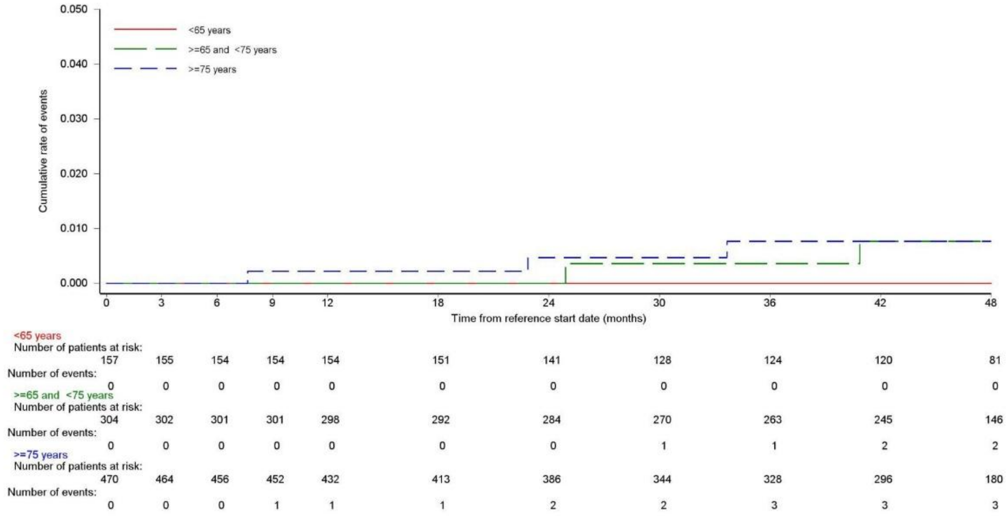

Figure S10. Kaplan-Meier plot for time-to-first occurrence of ICH by age at baseline for patients enrolled in Spain and Portugal (full analysis set)

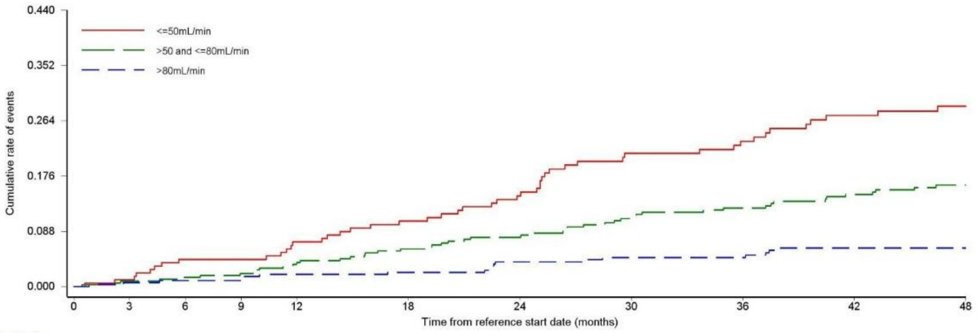

**<=50mL/min**											
Number of patients at risk:	184	182	176	176	167	160	146	126	119	102	60
Number of events:	0	2	8	8	13	19	26	37	40	46	48
**>50 and <=80mL/min**											
Number of patients at risk:	341	336	333	331	320	304	289	266	255	235	145
Number of events:	0	3	5	7	13	20	26	36	40	46	50
**>80mL/min**											
Number of patients at risk:	309	306	305	305	302	298	285	264	258	247	150
Number of events:	0	2	3	3	6	7	12	14	14	18	18

Figure S11. Kaplan-Meier plot for time-to-death for any cause by renal function at baseline for patients enrolled in Spain and Portugal (full analysis set)

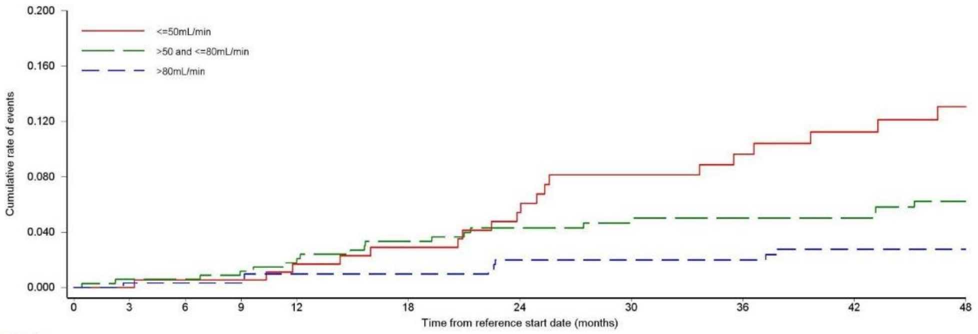

**<=50mL/min**											
Number of patients at risk:	184	182	176	176	167	160	146	126	119	102	60
Number of events:	0	0	1	1	3	5	9	13	15	17	19
**>50 and <=80mL/min**											
Number of patients at risk:	341	336	333	331	320	304	289	266	255	235	145
Number of events:	0	2	2	4	7	11	14	16	16	16	19
**>80mL/min**											
Number of patients at risk:	309	306	305	305	302	298	285	264	258	247	150
Number of events:	0	1	1	1	3	3	6	6	6	8	8

Figure S12. Kaplan-Meier plot for time-to-cardiovascular death by renal function at baseline for patients enrolled in Spain and Portugal (full analysis set)

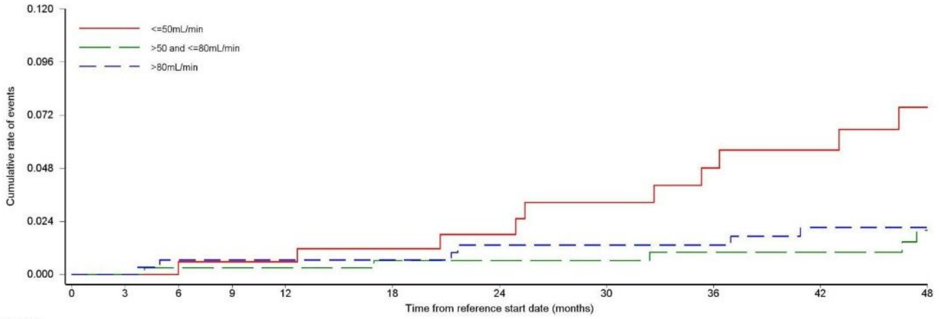

	0	3	6	9	12	18	24	30	36	42	48
**<=50mL/min**											
Number of patients at risk:	184	182	175	175	166	158	144	125	119	102	60
Number of events:	0	0	1	1	1	2	3	5	7	8	10
**>50 and <=80mL/min**											
Number of patients at risk:	341	336	332	330	319	302	287	264	252	232	141
Number of events:	0	0	1	1	1	2	2	2	3	3	5
**>80mL/min**											
Number of patients at risk:	309	306	303	303	300	296	282	261	255	243	146
Number of events:	0	0	2	2	2	2	4	4	4	6	6

Figure S13. Kaplan-Meier plot for time-to-first occurrence of stroke by renal function at baseline for patients enrolled in Spain and Portugal (full analysis set)

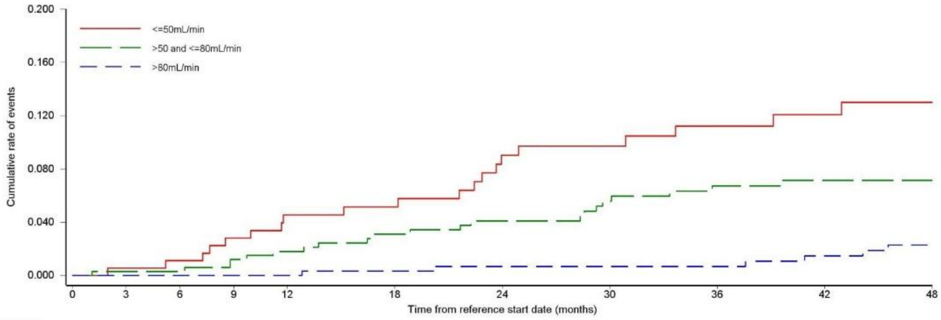

	0	3	6	9	12	18	24	30	36	42	48
**<=50mL/min**											
Number of patients at risk:	184	181	175	172	160	154	137	121	113	96	57
Number of events:	0	1	2	5	8	9	15	16	18	19	20
**>50 and <=80mL/min**											
Number of patients at risk:	341	336	333	328	315	295	277	252	239	220	133
Number of events:	0	1	1	4	6	10	13	17	20	21	21
**>80mL/min**											
Number of patients at risk:	309	306	305	305	302	297	283	263	257	245	147
Number of events:	0	0	0	0	0	1	2	2	2	4	6

Figure S14. Kaplan-Meier plot for time-to-first major bleeding event by renal function at baseline for patients enrolled in Spain and Portugal (full analysis set)

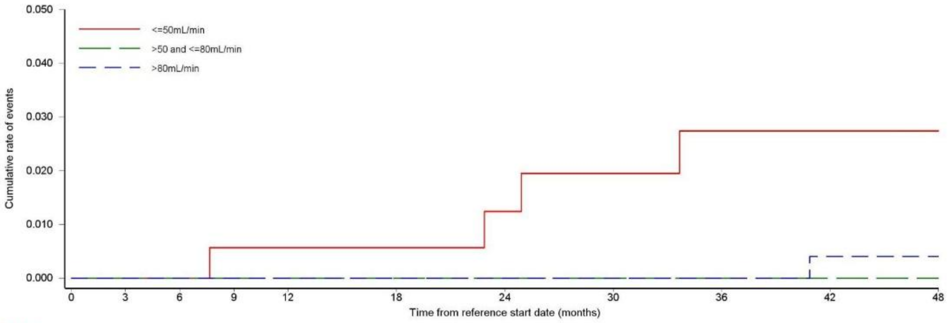

	0	3	6	9	12	18	24	30	36	42	48
**<=50mL/min**											
Number of patients at risk:	184	182	176	175	166	159	145	125	118	101	59
Number of events:	0	0	0	1	1	1	2	3	4	4	4
**>50 and <=80mL/min**											
Number of patients at risk:	341	336	333	331	320	304	289	266	255	235	145
Number of events:	0	0	0	0	0	0	0	0	0	0	0
**>80mL/min**											
Number of patients at risk:	309	306	305	305	302	298	285	264	258	246	149
Number of events:	0	0	0	0	0	0	0	0	0	1	1

Figure S15. Kaplan-Meier plot for time-to-first occurrence of ICH by renal function at baseline for patients enrolled in Spain and Portugal (full analysis set)

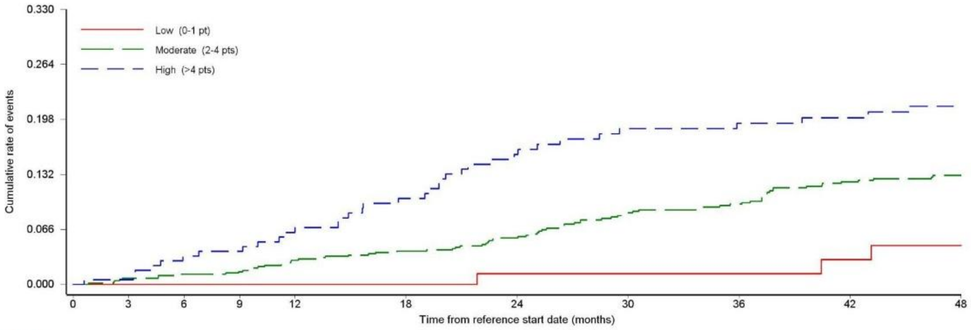

	0	3	6	9	12	18	24	30	36	42	48
**Low (0-1 pt)**											
Number of patients at risk:	83	82	82	82	82	79	71	65	60	57	37
Number of events:	0	0	0	0	0	0	1	1	1	2	3
**Moderate (2-4 pts)**											
Number of patients at risk:	657	650	646	644	627	613	587	536	518	477	288
Number of events:	0	5	8	10	19	26	36	54	60	75	79
**High (>4 pts)**											
Number of patients at risk:	176	175	170	169	163	153	142	130	126	117	75
Number of events:	0	1	6	7	12	18	27	32	33	34	36

Figure S16. Kaplan-Meier plot for time-to-death for any cause by CHA2DS2-VASc Risk Group (derived) at baseline for patients enrolled in Spain and Portugal (full analysis set)

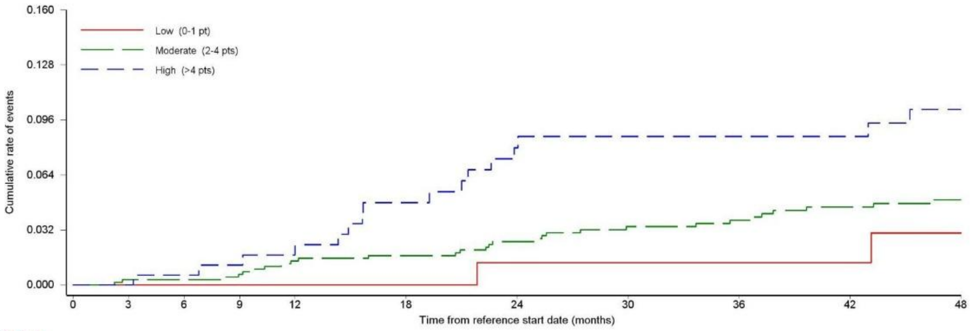

	0	3	6	9	12	18	24	30	36	42	48
**Low (0-1 pt)**											
Number of patients at risk:	83	82	82	82	82	79	71	65	60	57	37
Number of events:	0	0	0	0	0	0	1	1	1	1	2
**Moderate (2-4 pts)**											
Number of patients at risk:	657	650	646	644	627	613	587	536	518	477	288
Number of events:	0	2	2	4	9	11	16	21	23	27	29
**High (>4 pts)**											
Number of patients at risk:	176	175	170	169	163	153	142	130	126	117	75
Number of events:	0	0	1	2	4	8	13	14	14	14	16

Figure S17. Kaplan-Meier plot for time-to-cardiovascular death by CHA2DS2-VASc Risk Group (derived) at baseline for patients enrolled in Spain and Portugal (full analysis set)

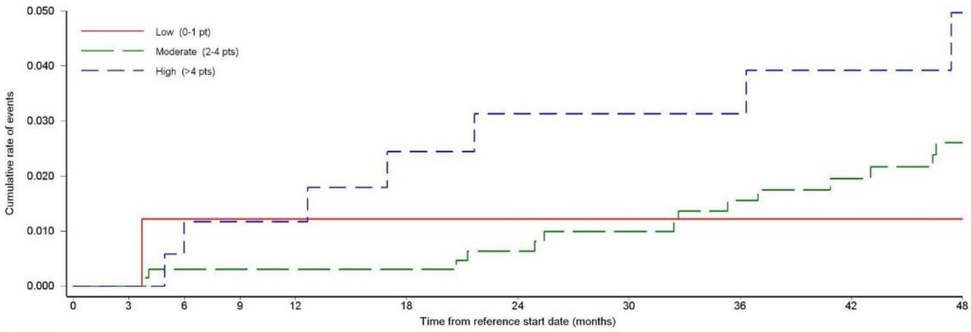

	0	3	6	9	12	18	24	30	36	42	48
**Low (0-1 pt)**											
Number of patients at risk:	83	82	81	81	81	78	71	65	60	57	37
Number of events:	0	0	1	1	1	1	1	1	1	1	1
**Moderate (2-4 pts)**											
Number of patients at risk:	657	650	644	642	625	611	584	533	514	472	284
Number of events:	0	0	2	2	2	2	4	6	9	11	14
**High (>4 pts)**											
Number of patients at risk:	176	175	168	167	161	149	137	126	123	114	71
Number of events:	0	0	2	2	2	4	5	5	5	6	7

Figure S18. Kaplan-Meier plot for time-to-first occurrence of stroke by CHA2DS2-VASc Risk Group (derived) at baseline for patients enrolled in Spain and Portugal (full analysis set)

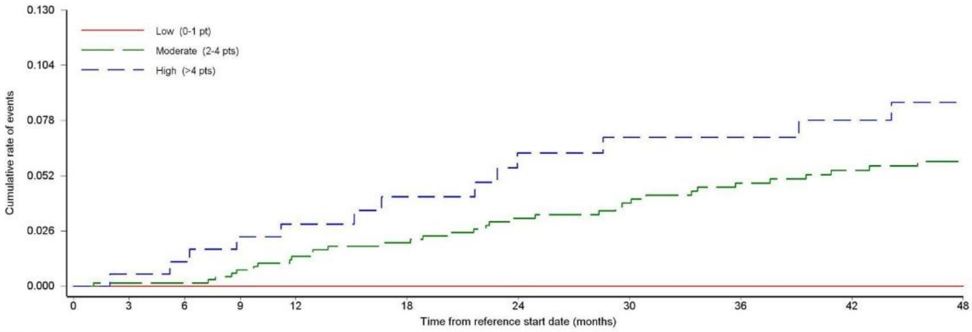

	0	3	6	9	12	18	24	30	36	42	48
**Low (0-1 pt)**											
Number of patients at risk:	83	82	82	82	82	79	71	65	60	57	37
Number of events:	0	0	0	0	0	0	0	0	0	0	0
**Moderate (2-4 pts)**											
Number of patients at risk:	657	650	646	640	619	603	571	521	500	460	276
Number of events:	0	1	1	5	9	13	20	24	29	32	34
**High (>4 pts)**											
Number of patients at risk:	176	174	169	166	159	147	135	125	121	111	69
Number of events:	0	1	2	4	5	7	10	11	11	12	13

Figure S19. Kaplan-Meier plot for time-to-first major bleeding event by CHA2DS2-VASc Risk Group (derived) at baseline for patients enrolled in Spain and Portugal (full analysis set)

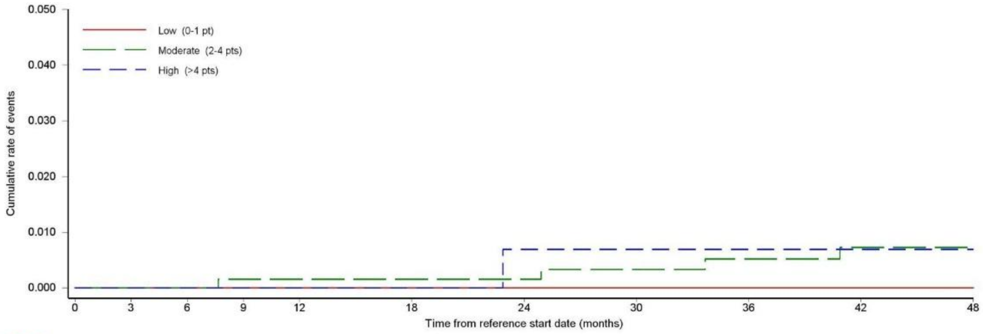

	0	3	6	9	12	18	24	30	36	42	48
**Low (0-1 pt)**											
Number of patients at risk:	83	82	82	82	82	79	71	65	60	57	37
Number of events:	0	0	0	0	0	0	0	0	0	0	0
**Moderate (2-4 pts)**											
Number of patients at risk:	657	650	646	643	626	612	586	535	517	475	286
Number of events:	0	0	0	1	1	1	1	2	3	4	4
**High (>4 pts)**											
Number of patients at risk:	176	175	170	169	163	153	142	130	126	117	75
Number of events:	0	0	0	0	0	0	1	1	1	1	1

Figure S20. Kaplan-Meier plot for time-to-first occurrence of ICH by CHA2DS2-VASc Risk Group (derived) at baseline for patients enrolled in Spain and Portugal (full analysis set)
